# The solution structures of native and patient monomeric human IgA1 reveal asymmetric extended structures: implications for function and IgAN disease

**DOI:** 10.1042/BJ20150612

**Published:** 2015-10-02

**Authors:** Gar Kay Hui, David W. Wright, Owen L. Vennard, Lucy E. Rayner, Melisa Pang, See Cheng Yeo, Jayesh Gor, Karen Molyneux, Jonathan Barratt, Stephen J. Perkins

**Affiliations:** * Department of Structural and Molecular Biology, Darwin Building, University College London, Gower Street, London WC1E 6BT, U.K.; † Department of Infection, Immunity and Inflammation, Maurice Shock Medical Sciences Building, University of Leicester, University Road, Leicester LE1 9HN, U.K.

**Keywords:** analytical ultracentrifugation, antibody, constrained modelling, human immunoglobulin A1 (IgA1), immunoglobulin A (IgA) nephropathy, neutron scattering, X-ray scattering

## Abstract

Native IgA1, for which no crystal structure is known, contains an O-galactosylated 23-residue hinge region that joins its Fab and Fc regions. IgA nephropathy (IgAN) is a leading cause of chronic kidney disease in developed countries. Because IgA1 in IgAN often has a poorly O-galactosylated hinge region, the solution structures of monomeric IgA1 from a healthy subject and three IgAN patients with four different O-galactosylation levels were studied. Analytical ultracentrifugation showed that all four IgA1 samples were monomeric with similar sedimentation coefficients, *s^0^_20,w_*. X-ray scattering showed that the radius of gyration (*R*_g_) slightly increased with IgA1 concentration, indicating self-association, although their distance distribution curves, *P(r)*, were unchanged with concentration. Neutron scattering indicated similar *R*_g_ values and *P(r)* curves, although IgA1 showed a propensity to aggregate in heavy water buffer. A new atomistic modelling procedure based on comparisons with 177000 conformationally-randomized IgA1 structures with the individual experimental scattering curves revealed similar extended Y-shaped solution structures for all four differentially-glycosylated IgA1 molecules. The final models indicated that the N-glycans at Asn^263^ were folded back against the Fc surface, the C-terminal tailpiece conformations were undefined and hinge O-galactosylation had little effect on the solution structure. The solution structures for full-length IgA1 showed extended hinges and the Fab and Fc regions were positioned asymmetrically to provide ample space for the functionally-important binding of two FcαR receptors to its Fc region. Whereas no link between O-galactosylation and the IgA1 solution structure was detected, an increase in IgA1 aggregation with reduced O-galactosylation may relate to IgAN.

## Abstract (precis)

Detailed analytical ultracentrifugation and X-ray/neutron scattering data and a new atomistic modelling approach revealed asymmetric extended solution structures for human IgA1 that account for its receptor-binding function. IgA1 with different hinge O-galactosylation patterns showed similar structures.

## INTRODUCTION

IgA is the most abundant antibody sub-class present on human mucosal surfaces, which themselves comprise the largest surface area in the human body exposed to pathogens [1]. Human IgA comprises two sub-classes, IgA1 and IgA^2^ and most IgA exist in monomeric or dimeric forms termed mIgA (monomeric IgA) and dIgA (dimeric IgA) respectively [2]. The main function of IgA is to act as the first line of defence in the genitourinary, respiratory and gastrointestinal tracts by preventing the entrance of pathogens into the body [1,2]. The upper respiratory and digestive tract secretions contain more IgA1 than IgA2 [1]. In serum, IgA is mainly monomeric and consists of approximately 90% IgA1 and 10% IgA2 [3], although the function of the two sub-classes remains unclear [2]. The effector functions of IgA are mediated by the Fc region, which binds the Fcα receptor (FcαR) to clear foreign antigens by opsonization and phagocytosis [1]. The complement system is weakly activated by IgA through its alternative and lectin pathways [4–7]. IgA nephropathy (IgAN) is the commonest pattern of glomerulonephritis in the world and an important cause of kidney failure with over 30% of patients progressing to end-stage renal disease within 20 years of diagnosis [8]. IgAN is characterized by the deposition of IgA1-containing immune complexes in the mesangium which triggers glomerular injury through activation of resident glomerular cells [9,10].

**Figure 1:**
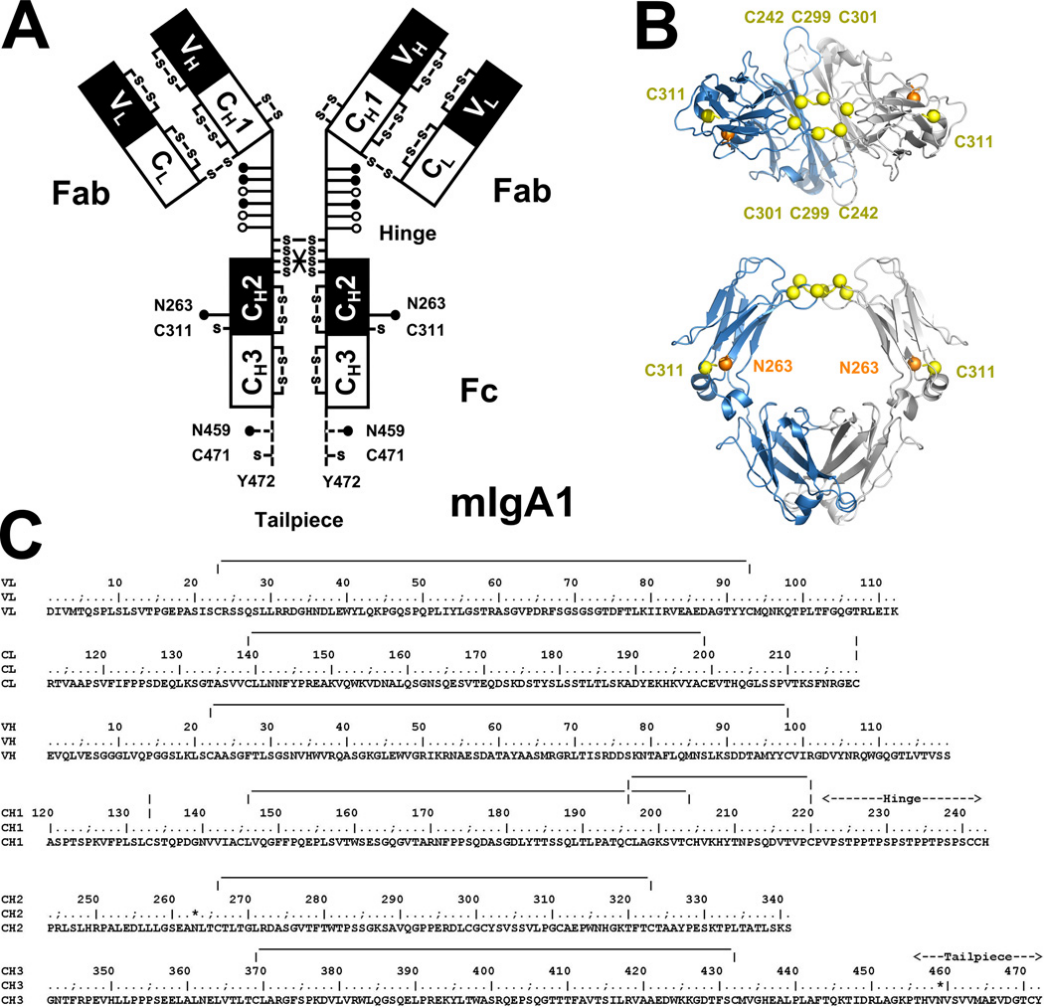
The human IgA1 domain structure (**A**) The schematic cartoon shows the heavy chains with variable and constant V_H_, C_H_1, C_H_2 and C_H_3 domains and the light chains with V_L_ and C_L_ domains. Interchain disulfide bridges stabilize the IgA1 structure. Two conserved N-glycosylation sites occurred at Asn^263^ and Asn^459^ (●). The hinge region between the Fab and Fc regions is composed of 21 residues (222-PVPSTPPTPSPSTPPTPSPSCC-242, anchored at Pro^221^ and His^243^), with the capacity to bind six O-linked glycans on each hinge (○ and ●). The sites at Thr^225^, Thr^228^ and Ser^232^ were assumed to be occupied for the present study (●; Experimental). An 18-residue tailpiece between Pro^455^ and Tyr^472^ (broken line) lies at the C-terminus of each heavy chain. (**B**) Ribbon diagrams of the IgA1 Fc region (PDB code 1OW0) in two views to show the intrachain disulfide bridges between two Cys^242^–Cys^299^ pairs at the top of the Fc region. Two unpaired Cys^301^ residues are shown also, which are probably bridged to Cys^241^ (result not shown). Also shown are Cys^311^ and the glycosylation site at Asn^263^. The two heavy chains are shown in grey and light blue. Another IgA1 Fc crystal structure (PDB code 2QEJ; not shown) revealed different disulfide bridges (two Cys^242^–Cys^301^ pairs and a Cys^299^–Cys^299^ pair). (**C**) The sequence of human IgA1 is shown (Experimental). Known disulfide bridges are indicated by vertical and horizontal lines. Asn^263^ and Asn^459^ are asterisked.

IgA1 and IgA2 primarily differ in their hinge region. IgA1 contains a 23-residue hinge between the Fab and Fc regions (Figure 1), which is replaced by a short 10-residue hinge in IgA2. The IgA1 hinge is rich in proline residues and serine/threonine residues, the latter having the capacity for binding six O-linked oligosaccharides on each of the two hinges. These six sites can be occupied by α1 O-linked *N*-acetylgalactosamine (GalNAc) residues which may be extended with the addition of galactose (Gal) and sialic acid (NeuNAc) in different combinations [11–14]. This O-galactosylation gives rise to a heterogeneous population of IgA1 molecules in an individual [12,15–17]. The O-galactosylation of the hinge has been implicated in IgAN, where the IgA1 deposits are often poorly galactosylated [18]. Numerous studies have postulated that the reduced level of O-linked IgA1 glycans in IgAN have a pathogenic role [9,19,20], however the effects of the altered O-galactosylation upon the 3D IgA1 structure and its function remain to be elucidated.

A molecular understanding of IgA1 function and disease requires an atomistic solution structure for IgA1, especially for its hinge region. There is no crystal structure for the full-length IgA1 antibody, a common issue with many antibodies and attributable to segmental flexibility at the hinge region that hinders crystal growth. Intact Y-shaped IgA structures were originally observed in electron micrographs [21–23]. An extended solution structure for human IgA1 was determined by constrained scattering modelling, this being the first such structure determination of this type [24]. The previous experimental data were derived from pooled serum monomer IgA1 and was therefore heterogeneous, and homology models and not crystal structures were used for the scattering fits that assumed 2-fold symmetry in intact IgA1 [24]. In the present study, the IgA1 solution structure was re-investigated in full. First, IgA1 was purified separately from four individuals to reduce its heterogeneity, but now targeting a broad range of O-galactosylation levels. Second, the use of size-distribution analyses *c(s)* by analytical ultracentrifugation, high-throughput X-ray instruments and new neutron scattering instruments with higher fluxes provide much enhanced data sets for modelling. Third, crystal structures for a human IgA1 Fab region [25], an IgA Fc region in complex with FcαR [26] and an IgA Fc region in complex with staphylococcal superantigen-like protein [27] permit more accurate structural modelling of intact IgA1. Fourth, although constrained scattering modelling has now been applied to 14 intact antibodies [28,29], this method is now much enhanced by new Monte Carlo methods that generate far larger libraries of randomized trial structures for improved curve fits [30,31]. These four developments enabled more accurate molecular modelling of more extensive scattering data sets for monomeric IgA1. We obtained new molecular insights on IgA1 receptor binding and on potential relationships between O-galactosylation levels in the IgA1 hinge and its solution structure and IgAN disease.

## EXPERIMENTAL

### Purification and composition of IgA1

Serum was obtained from one healthy subject and three patients (denoted 2, 3 and 4) with biopsy-proven IgAN who attended the John Walls Renal Unit, Leicester General Hospital. At the time of serum collection, all participants had normal renal function (estimated glomerular filtration rate > 90 ml/min). All healthy subject and patient personal data were anonymized. The study was approved by the Leicestershire, Northamptonshire and Rutland Research Ethics Committee and all subjects gave written informed consent. The four IgA1 antibodies were each purified from serum by affinity chromatography using jacalin-linked agarose (Vector Laboratories) [32] and dialysed against PBS (137 mM NaCl, 8.1 mM Na_2_HPO_4_, 2.7 mM KCl, 1.5 mM KH_2_PO_4_, pH 7.4) overnight at 4°C. Monomeric IgA1 was isolated from the total IgA1 preparation by FPLC chromatography using a Superdex 200 pg column (Amersham Biosciences), concentrated and stored at −20°C until further analysis. Immediately prior to experiments, each IgA1 sample was further purified by gel filtration using a Superose 6 column (Amersham Biosciences) to remove non-specific aggregates, then concentrated using Amicon Ultra spin concentrator (50 kDa molecular mass cut-off) and dialysed at 4°C against the appropriate measurement buffer. Prior to measurements, IgA1 samples were stored at 4°C and were not frozen in order to avoid aggregate formation.

The relative hinge region O-galactosylation of the four IgA1 samples was determined using an IgA1-specific enzyme-linked *Helix aspersa* (HAA) lectin-binding assay with specificity for O-linked GalNAc without Gal attached [19,32,33]. Serum IgA was captured on to immunoplates (Nunc Life Technologies) using anti-human IgA antibody (Dako) and overnight incubation at 4°C. The captured IgA was incubated overnight with 75 milliunits/ml neuraminidase (New England Biolabs UK Ltd) in sodium acetate buffer, pH 5, to remove terminal NeuNAcs residues. The desialylated IgA1 was incubated with biotinylated HAA lectin (Sigma–Aldrich, diluted 1/500 in PBS) for 90 min at room temperature. After washing, the samples were incubated with peroxidase-conjugated avidin (Vector Laboratories; diluted 1/2000 in PBS). Lectin binding was measured colorimetrically using the substrates 1,2-phenylenediamine dihydrochloride and hydrogen peroxide on an ELISA plate reader (Titertek Multiskan) using a 492 nm ultraviolet filter. Results were expressed as *D* values, normalized to serum samples with known haemagglutinin (HA)-lectin binding. In this assay, an increase in HAA lectin binding to IgA1 indicated an increase in exposed O-linked GalNAc and corresponded to reduced hinge O-galactosylation. The samples were analysed independently in triplicate and results reported as the mean and the S.E.M.

The human monomeric IgA1 amino acid sequence [24] (Figure 1C) provided the molecular mass, unhydrated and hydrated volumes, partial specific volume and absorption coefficient for the experimental analyses. As defined in Figure 1, the sequences of the V_H_ and V_L_ variable regions were taken from the crystal structure for a human IgA Fab region (PDB codes 3M8O) [34] and the constant region sequences C_H_1, C_H_2, C_H_3 and C_L_ were taken from our previous analysis (Uniprot code P01876) [24]. The IgA1 samples from the healthy subject and three IgAN patients were polyclonal and therefore contain heterogeneous complementarity determining regions in the V_H_ and V_L_ domains [15,16]. The IgA1 molecular mass was calculated as 164 kDa, its unhydrated volume was 205.7 nm^3^, its hydrated volume was 272.5 nm^3^ (based on a hydration of 0.3g of water per gram of glycoprotein and an electrostricted volume of 0.0245 nm^3^ per bound water molecule), its partial specific volume ν was 0.724 ml/g and its absorption coefficient at 280 nm was 12.7 (1%, 1 cm path-length) [24,35,36]. All data were recorded in PBS in light or heavy water at 20°C. The PBS buffer densities of 1.00543 g/ml in light water and 1.11238 g/ml in 100% ^2^H_2_O at 20°C and the buffer viscosity of 0.01019 cp were calculated using SEDNTERP [37].

### Experimental data collection for IgA1

Analytical ultracentrifugation data were obtained on two Beckman XL-I instruments equipped with AnTi50 and AnTi60 rotors. Sedimentation velocity data were acquired for all four IgA1 samples at 20°C in H_2_O. Healthy subject IgA1 was studied at concentrations of 0.19, 0.32, 0.54, 0.90, 1.51 and 2.52 mg/ml; patient 2 IgA1 was studied at concentrations of 0.41, 0.69, 1.15 and 1.93 mg/ml; patient 3 IgA1 was studied at concentrations of 0.23, 0.39, 0.66 and 1.11 mg/ml; and patient 4 IgA1 was studied at concentrations of 0.41, 0.68 and 1.13 mg/ml. Data were collected at rotor speeds of 20000, 30000, 40000 and 50000 rpm in two-sector cells with column heights of 12 mm. Sedimentation analysis was performed using direct boundary Lamm fits of up to 150 scans using SEDFIT (version 14.1) [38,39]. SEDFIT gave size-distribution analyses *c(s)* that revealed the sedimentation species; this assumed these species to have the same frictional ratio *f/f_0_*, where *f* is the frictional coefficient of the macromolecule and *f_0_* is the frictional coefficient of the sphere with the same hydrated volume as the macromolecule. The final SEDFIT analyses used a fixed resolution of 200 and optimized the *c(s)* fit by floating *f/f_0_*, the baseline, the meniscus and the bottom of the cell until the overall RMSDs and visual appearance of the fits were satisfactory. The percentage of oligomers in the total loading concentration was derived using the *c(s)* integration function.

X-ray scattering data were obtained at 20°C for the IgA1 samples in light water in a single beam session in four-bunch mode on Instrument ID02 at the European Synchrotron Radiation Facility, operating with a ring energy of 6.0 GeV [40]. A sample-to-detector distance of 3.0 m was used. Data were acquired using a fast readout low noise camera (FreLoN) with a resolution of 512×512 pixels. Healthy subject IgA1 was studied at concentrations of 0.25, 0.51, 0.76 and 1.02 mg/ml; patient 2 IgA1 was studied at 0.16, 0.33, 0.49, 0.66, 0.99 and 1.33 mg/ml; patient 3 IgA1 was studied at 0.12, 0.24, 0.36, 0.48, 0.72 and 0.97 mg/ml; patient 4 IgA1 was studied at 0.15, 0.31, 0.47, 0.63, 0.94 and 1.26 mg/ml. This totalled 88 samples. Sample volumes of 100 μl were measured four times in a polycarboxylate capillary at 2-mm diameter that avoids protein deposits during exposures, with the samples being moved continuously during beam exposure to reduce radiation damage. Thus data sets of 10 time-frames, with a frame exposure time of 0.1 or 0.2 s each, were acquired in quadruplicate as a subject of reproducibility. Online checks during data acquisition confirmed the absence of radiation damage, after which each set of 10 frames were averaged.

Neutron scattering data for IgA1 in heavy water buffer were obtained on Instrument SANS2D at the pulsed neutron source ISIS at the Rutherford Appleton Laboratory [41]. A pulsed neutron beam was derived from proton beam currents of approximately 40 μA. SANS2D data were recorded with 4 m of collimation, a 4-m sample-to-detector distance, a 12-mm beam diameter and a wavelength range from 0.175 to 1.65 nm obtained using TOF methods. Samples were measured in circular banjo cells with 2 mm thickness positioned in a thermostatted sample rack set at 20°C. Data was collected for healthy subject IgA1 at concentrations of 0.43, 0.85 and 1.7 mg/ml, patient 2 IgA1 at 0.6, 1.2, 1.8 and 2.4 mg/ml, patient 3 IgA1 at 0.5, 1.0, 1.6, 2.2 mg/ml and patient 4 IgA1 at 0.43 and 0.86 mg/ml.

For a given solute–solvent contrast, the radius of gyration (*R*_g_) is a measure of structural elongation if the internal inhomogeneity of scattering densities within the protein has no effect. Guinier analyses of the scattering curve *I(Q)* at low scattering vectors *Q* (where *Q*=4π sin θ/λ; 2*θ* is the scattering angle and *λ* is the wavelength) gives the *R*_g_ and the forward scattering at zero angle *I*(0) [42]:

$$\ln I\;\left(Q\right)=\ln I\left(0\right)-\frac{{R_{g}}^{2}Q^{2}}{3}\;$$

This expression is valid in a *Q·R*_g_ range up to 1.5. If the structure is elongated, the mean *R*_g_ of cross-sectional structure *R*_xs_ and the mean cross-sectional intensity at zero angle [*I*(*Q*)*Q*]*_Q_*_→0_ is obtained from:

$$\ln\;\left[I\left(Q\right)Q\right]={\left[I\left(Q\right)Q\right]}_{Q\to 0}\;-\;\;\frac{{R_{\mathrm{xs}}}^{2}Q^{2}}{2}$$

The cross-sectional plot for immunoglobulins exhibits two distinct regions, a steeper innermost one and a flatter outermost one [43]. The two analyses are denoted as *R*_xs-1_ and *R*_xs-2_ respectively. The *R*_g_ and *R*_xs_ analyses were performed using an interactive PERL script program SCTPL7 (J. T. Eaton and S. J. Perkins, unpublished software) on Silicon Graphics OCTANE Workstations. Indirect Fourier transformation of the scattering data *I(Q)* which was measured in reciprocal space into real space to give the distance distribution function *P(r)* was carried out using the program GNOM [44]:

$$P\;\left(r\right)=\;\frac{1}{2\pi^{2\;}}\;\int_{0}^{\infty }I\left(Q\right)Qr\sin\left(Qr\right)dQ$$

*P(r)* corresponds to the distribution of distances *r* between volume elements. This provides the maximum dimension *L* of IgA1 and its most commonly occurring distance vector *M* in real space. For this, the X-ray *I(Q)* curve utilized up to 441 data points in the *Q* range between 0.13 and 2.10 nm^−1^. The neutron *I(Q)* curve utilized up to 45 data points in the *Q* range between 0.18 and 1.6 nm^−1^.

### Generation of a starting structural model for PTerm455

The modelling of IgA1 was initiated by two crystal structures for the Fab and Fc regions of IgA (PDB codes 3M8O and 1OW0 respectively) [26,34]. The initial scattering data used for modelling was that for PTerm455, a tailpiece-deleted recombinant IgA1 with its C-terminus at Pro^455^ (Figure 1C) [24]. Based on these two crystal structures, the starting PTerm455 conformation (termed ‘assembled’) was constructed as follows.

(i) The full hinge was defined as the 21-residue segment 222-VPSTPPTPSPSTPPTPSPSCC-242, anchored at its flanking residues Pro^221^ and His^243^. Two distinct hinge structures were modelled using PyMOL v1.3r1 (Schrodinger) that created Y-shaped and T-shaped PTerm455 structures. The Y-shaped structure was generated using the PyMOL build_seq script (PyMOL Script Repository, Queen's University) with backbone ϕ and ψ angles of 10°. The T-shaped structure was generated from our previous PTerm455 structure [24].

(ii) The C-terminal Fc pentapeptide was defined as 451-LAGKP-455 and added to the Fc structure using the PyMOL build_seq script.

(iii) The Asn^263^ N-glycan was taken to be biantennary with both branches having the same monosaccharides [45]. Because only one β1→6-linked NeuNAc.Gal.GlcNAc branch was visible in the Fc crystal structure, its conformation was copied to form a second β1→3-linked branch using PyMOL, to give a structure of NeuNAc_2_.Gal_2_.GlcNAc_2_.Fuc.Man_3_.GlcNAc_2_. The appropriate CONECT records were added for all the residues and the glycosidic bond.

This ‘assembled’ PTerm455 structure was prepared for energy minimization through the addition of H atoms and the optimization of steric overlaps within the crystal structure using the glycan reader component of CHARMM-GUI [46,47] and the CHARMM36 force field [48–52]. Disulfide bridges were included between Cys^23^–Cys^93^ and Cys^139^–Cys^199^ in each light chain and Cys^22^–Cys^98^, Cys^145^-Cys^204^, Cys^196^-Cys^220^, Cys^266^-Cys^323^ and Cys^369^-Cys^432^ in each heavy chain. Interchain disulfide bridges were formed between Cys^219^ in the C_L_ domain and Cys^133^ in the C_H_1 domain and two between Cys^299^ in the C_H_2 domain and Cys^242^ in the hinge (Figure 1B). Two thousand steps of energy minimization was performed on both Y-shaped and T-shaped structures in generalized Born implicit solvent using the NAMD2 software for MD simulations to create two structurally varied PTerm455 structures (termed ‘initial’) [53,54].

### Generation of trial structural models for full-length IgA1

The four main conformational unknowns in the full-length IgA1 structure were each considered as follows.

(i) The Asn^263^ N-glycan conformations (NGs) were investigated by a NAMD2 MD simulation of the ‘initial’ T-shaped PTerm455 structure as above, in which the protein backbone was constrained to maintain the same conformation using a force of 4 kcal mol^−1^ Å^−2^ (1 cal ≡ 4.184 J). The protein backbone was held fixed in this. The system was heated from 50 K to 300 K over 320 ps before an equilibration run of 1 ns. To accelerate the sampling of randomized N-glycan conformations, the system was heated from 300 K to 750 K over 580 ps and a further 2 ns run was performed. Analysis of the glycan structures sampled during the simulation identified two distinct non-crystal like conformers. Consequently, a library of three N-glycan structures, denoted NG0–NG2, was produced to be added symmetrically to both Fc chains in all global PTerm455 models.

(ii) Trial hinge conformations in the ‘initial’ PTerm455 structure were computed using the Monte Carlo simulation module in SASSIE [31]. The backbone ϕ and ψ angles of the 20 peptides in the 21-residue hinge (222-VPSTPPTP-SPSTPPTPSPSCC-242) and the four peptides in the five-residue C-termini (451-LAGKP-455) were randomized independently of one another. Monte Carlo runs for each of the ‘initial’ Y-shaped and T-shaped PTerm455 models used maximum ϕ and ψ rotational steps of up to 10° and 30° respectively from the previous values, giving 57611 conformations. Each of the three N-glycan conformations NG0-NG2 from (i) above were attached to the 57611 conformations using SASSIE SASmol scripts [31] to give 172833 trial PTerm455 models. PTerm455 curve fits based on an *R* factor filter of less than or equal to 7% reduced this total to 36621 models for the next modelling stage.

(iii) The C-terminal tailpiece 456-THVNVSVVMAEVDG-TCY-472 and an Asn^459^ N-glycan were added to the ‘initial’ T-shaped PTerm455 model to create the full-length IgA1 structure (Figure 1A). This was achieved with the PyMOL build_seq script. A standard biantennary glycan was used for Asn^459^ [24,45], making a total of 2×28 amino acid and glycan residues. The resulting full-length IgA1 structure was processed using the CHARMM-GUI glycan reader as above. In the MD simulation, the IgA1 structure was energy minimized using NAMD2 for 2000 steps before being heated from 50 K to 300 K over 320 ps. After this, an equilibration run of 1 ns was performed, followed by a further 2 ns of production simulation. In all simulations, the protein backbone was constrained to remain close to the starting structure using a force of 4 kcal mol^−1^ Å^−2^ except for the C-terminal tailpiece 451-LAGKPTHVNVSVVMAEVDGTCY-472 which was allowed to move freely. Principal component analysis using the Bio3D package [55] identified four distinct tailpiece structures TP1–TP4, each of which were superimposed upon the 36621 PTerm455 models using the main chain atoms of Arg^450^ to give 146484 full-length IgA1 models.

(iv) Six NeuNAc.Gal.GalNAc O-glycans were added to the two hinges of the 146484 IgA1 models, these being representative of reported O-glycan structures [56,57]. They were located at Thr^225^, Thr^228^ and Ser^232^ as the most highly populated sites of the six potential sites at Thr^225^, Thr^228^, Ser^230^, Ser^232^, Thr^233^ and Thr^236^ [17,56,57]. This was achieved by overlapping the backbone and β-carbon atoms of the linked serine/threonine residue connected to an extended O-linked trisaccharide conformation using PyMOL.

### Scattering curve calculation using SCT

Scattering curves were calculated using the recently-released open source SCT software suite [58]. Unhydrated scattering curves were computed from the 172833 trial PTerm455 models for comparison with the PTerm455 neutron scattering curve in heavy water [24]. Hydrated scattering curves were computed from the 146484 trial full-length IgA1 models for comparison with the experimental X-ray scattering data. The atomic co-ordinates were coarse grained into small sphere models using a grid with cube-side length of 0.530 nm and a cut-off of four atoms; these parameters were optimized by SCT to reproduce the unhydrated protein volume. The hydration shell corresponding to 0.3 g of water per gram of protein was created by adding hydration spheres [35,58,59]. Scattering curves *I(Q)* were calculated using the Debye equation adapted to spheres [60]. Each experimental *I(Q)* value was matched to the theoretical calculated *I(Q)* value with the closest *Q* value, after which the *R* factor was computed by analogy with crystallography where lower *R* factors represent better fits:

$$R_{\mathrm{factor}}\;=\;\frac{\sum \left\|∥I_{Expt}\left(Q\right)∥-\eta ∥I_{Theor}\left(Q\right)∥\right\|}{\sum ∥I_{Expt}\left(Q\right)∥}\times 100$$

*η* is a scaling factor used to match the theoretical curve to the experimental *I(0)* value. An iterative search to minimize the *R* factor was used to determine *η*. This procedure was applied twice, namely to the 172833 PTerm455 models to analyse the N-glycan conformations NG0–NG2 and the 146484 full-length IgA1 models to analyse the four tailpiece conformations TP1–TP4. Principal component analysis using the Bio3D package [55] identified four distinct clusters of full-length IgA1 structures FL1–FL4. A representative best-fit atomistic model and all 112 models of the FL3 cluster are available from Supplementary Materials. The best-fit FL1, FL2, FL3 and FL4 structures corresponded to frames 51, 46, 62 and 52 of the 54, 152, 112 and 153 structures respectively in these files.

## RESULTS

### Purification and characterization of four IgA1 samples

**Figure 2:**
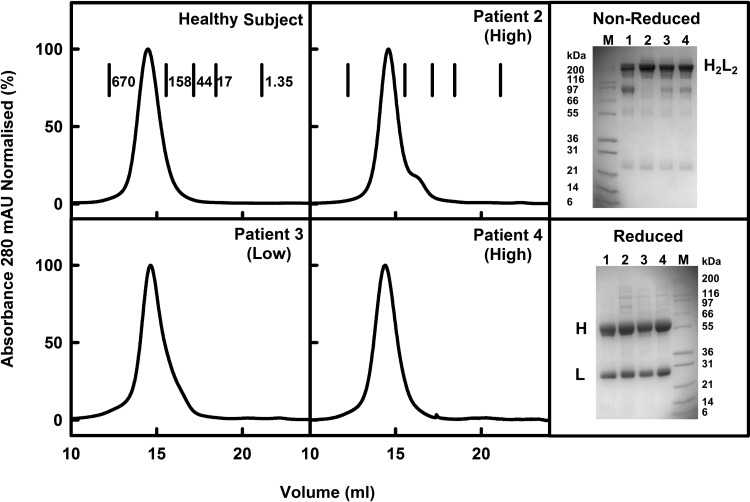
Purification of human IgA1 The elution of monomeric IgA1 from the healthy subject and three IgAN patients from a Superose 6 10/300 gel filtration column is shown in the four left panels (mAU, milli-absorbance units), together with molecular mass markers (kDa). The non-reduced and reduced SDS/PAGE analyses of IgA1 are shown in the two right panels with H representing the heavy chain and L representing the light chain. M denotes the molecular mass markers. Lanes 1–4 represent the healthy subject and patients 2, 3 and 4 respectively. Patients with high and low HAA lectin-binding values are labelled as ‘high’ and ‘low’ respectively here and in Figures 3–6 below.

Four individuals were selected to permit the study of a wide range of O-galactosylation contents in IgA1. One healthy subject and three patient IgA1 samples were purified from their serum using lectin jacalin affinity chromatography (Experimental), followed by gel filtration immediately prior to the ultracentrifugation or scattering experiments to ensure that IgA1 was monodisperse. IgA1 was found to aggregate if stored frozen. In all four cases, IgA1 eluted as a principally symmetric main peak at 14.5 ml (Figure 2). These showed a single band between 200 and 116 kDa in non-reducing SDS/PAGE that corresponds to the expected masses of ∼160 kDa for intact IgA1 [61]. Under reducing conditions, the heavy chains migrated at between 55 and 66 kDa and the light chains migrated at between 21.5 and 31 kDa. These bands corresponded to the α-heavy chain of ∼62 kDa and *κ*/*λ* light chains of ∼30 kDa as expected (Figure 2). The HAA lectin-binding assay (Experimental) revealed that the healthy subject IgA1 had a median hinge O-galactosylation profile (HAA lectin binding=1.11 mean *D*_492_; S.E.M. ± 0.04), whereas IgAN patients 2 and 4 displayed poor hinge region O-galactosylation (high HAA lectin binding=1.18 and 1.29 mean *D*_492_ respectively; both S.E.M ± 0.04) and patient 3 showed a low HAA lectin binding (1.00 mean *D*_492_; S.E.M. ± 0.03) that corresponded to more extensive O-linked galactosylation. Changes in the pattern of IgA1 O-glycosylation are the most consistently observed pathological feature in populations of IgAN patients from North America, Europe and Asia; however, there is a broad spread in O-glycosylation levels. Patients 2 and 4 showed lower levels of O-galactosylation which is more commonly observed in IgAN, whereas patient 3 showed a less common higher O-galactosylation which predisposes to non-progressive IgAN disease. The four IgA1 samples thus displayed a broad range of O-glycan contents.

### Analytical ultracentrifugation of the IgA1 samples

**Figure 3:**
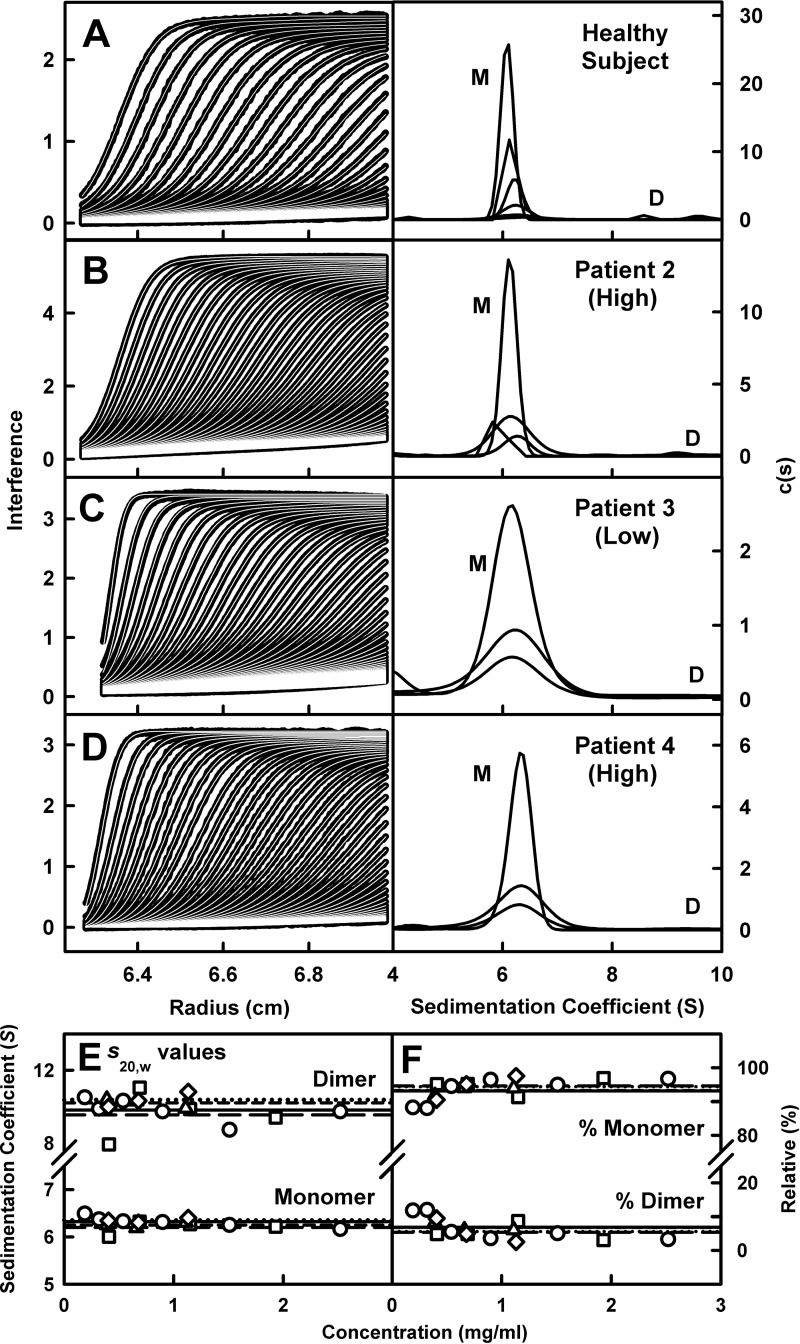
Sedimentation velocity analyses of IgA1 The experimentally observed sedimentation boundaries for IgA1 samples from (**A**) the healthy subject and (**B–D**) patients 2, 3 and 4 in PBS-137 were recorded at a rotor speed of 30000 rpm and 20°C. Approximately 30 boundaries (black outlines) are shown for up to 150 scans at intervals of about every fifth scan for clarity, fitted using SEDFIT as shown (thin white lines). The right panel shows the size-distribution analyses *c(s)*, revealing a monomer (M) peak at *s_20,w_* values of 6.2–6.3 S for the four IgA1 samples. Small amounts of a dimer (D) were detectable at ∼9 S. (**E**) The *s_20,w_* values for monomeric and dimeric IgA1 are shown as a function of IgA1 concentration (healthy subject, ○, —; patient 2, □, − −; patient 3, △, - - -; patient 4, ⋄, ···). (**F**) The percentages of monomer and dimer in each IgA1 sample from integration of the *c(s)* analyses are shown.

Sedimentation velocity experiments examined the shape and mass of the four IgA1 samples. The SEDFIT analyses of 17 separate IgA1 runs in concentration ranges between 0.19 and 2.52 mg/ml in PBS buffer (Experimental) involved fits of as many as 150 scans and the good agreement between the experimental boundary scans and fitted lines is clear (left panels; Figure 3). The size distribution analyses *c(s)* showed that all four IgA1 samples were predominantly monomeric in solution and all were accompanied by a minor dimer peak. The monomer peak was observed at *s^0^_20,w_* values of 6.2–6.4 S for all four IgA1 samples. These *s^0^_20,w_* values were consistent with previously reported values of 6.1–7.0 S for human IgA1 [24,45,61,62]. The IgA1 sedimentation rates did not depend on the concentration (Figure 3E). For 1.51 mg/ml healthy subject IgA1, the monomer *s^0^_20,w_* values were similar at 6.27 S for 20000 rpm, 6.25 S for 30000 rpm and 6.20 S for 40000 rpm, thus almost no dependence on rotor speed was seen that would otherwise imply the existence of very flexible hinge regions. All the IgA1 data reported in this study were for 30000 rpm. The average *s_20,w_* values of the IgA1 monomer were 6.32±0.11 S (healthy subject), 6.20±0.14 S (patient 2), 6.25±0.06 S (patient 3) and 6.35±0.05 S (patient 4; Table 1A). The *s^0^_20,w_* values of IgA1 were similar within error showing that IgA1 is stable and reproducible in shape for all four samples. The *c(s)* analyses indicated that the average molecular masses of the monomer IgA1 peaks were 168±12 kDa (healthy subject), 153±24 kDa (patient 2), 174±4 kDa (patient 3) and 163±20 kDa (patient 4) in PBS in H_2_O. These agreed well with the expected molecular mass of IgA1 of ∼160 kDa [62], although that for IgA1 from patient 3 was slightly higher. Integration of the monomer and dimer *c(s)* peaks showed high amounts of monomeric IgA1 with mean values of 93.2% (healthy subject), 94.7% (patient 2), 93.2% (patient 3) and 94.4% (patient 4; Figure 3F). No concentration dependence was detected in Figures 3(E) and 3(F).

**Table 1:** Experimental X-ray and neutron scattering and sedimentation coefficient data for human IgA1

	Extrapolated *R*_g_ (nm)	*R*_g_ range (nm)*	Mean *R*_xs-1_ (nm)*	Mean *R*_xs-2_ (nm)*	*L* (nm)	*s^0^_20,w_* (S)^†^
(A) X-ray data^‡^						
Healthy subject (14 curves)	5.96	5.93–6.30	2.46±0.06	1.57±0.07	21.2±1.3	6.32±0.11
Patient 2 (21 curves)	5.92	5.58–6.52	2.50±0.06	1.55±0.04	22.1±1.6	6.20±0.14
Patient 3 (19 curves)	5.93	5.70–6.32	2.46±0.05	1.58±0.07	21.3±1.5	6.29±0.10
Patient 4 (22 curves)	5.92	5.77–6.44	2.46±0.04	1.54±0.05	21.0±1.4	6.35±0.05
Overall average^§^	5.93±0.02	n.a.	2.48±0.08	1.58±0.13	21.2±1.5	6.29±0.11
(B) Neutron data*						
Healthy subject (3 curves)	n.a.	6.10±0.26	1.99±0.06	1.37±0.06	20.9±5.1	n.a.
Patient 3 (4 curves)	n.a.	6.36±0.23	2.25±0.08	1.49±0.06	18.6±1.3	n.a.
Overall average^§^	n.a.	6.25±0.26	2.13±0.15	1.44±0.08	19.6±3.3	n.a.

*The range of X-ray values observed from the Guinier *R_g_* analyses is shown. In all other cases, the mean *R_g_, R_xs-1_* and *R_xs-2_* values are shown.

†The mean *s^0^_20,w_* values from the ultracentrifugation experiments at 30,000 rpm are shown.

‡The 76 experimental X-ray scattering curves exclude the concentrations below 0.25 mg/ml that were not used in the extrapolations to zero concentration (Experimental).

§The overall averages from all the experimental data sets are shown.

n.a., not applicable.

The minor dimer peak seen in the *c(s)* analyses occurred at *s^0^_20,w_* values between 8 S and 12 S (Figure 3E). The *s^0^_20,w_* values of the IgA1 dimer were 9.82±0.62 S (healthy subject), 9.56±1.29 S (patient 2), 10.21±0.26 S (patient 3) and 10.38±0.41 S (patient 4) in PBS at 20°C. Although small in size and not accurately measured, this peak occurred at mean molecular masses of 326±33 kDa (healthy subject), 288±40 kDa (patient 2), 364±15 kDa (patient 3) and 338±30 kDa (patient 4). These masses are consistent with the formation of small amounts of an IgA1 dimer from the non-covalent association of two monomers with an expected mass of ∼320 kDa. The minor amount of dimer did not detectably alter with concentration and amounted to approximately 3%–5% or less of the total IgA1 content (Figure 3F).

### X-ray and neutron scattering of the IgA1 samples

X-ray scattering with light water buffer monitored the shape of the hydration shell surrounding IgA1, as well as its overall antibody structure, whereas neutron scattering with heavy water buffer monitored the overall shape of the unhydrated structure because the hydration shell was largely invisible in this buffer [59]. The heavy water buffer also monitored potential IgA1 self-association, because heavy water is associated with weaker solute–solvent hydration.

**Figure 4:**
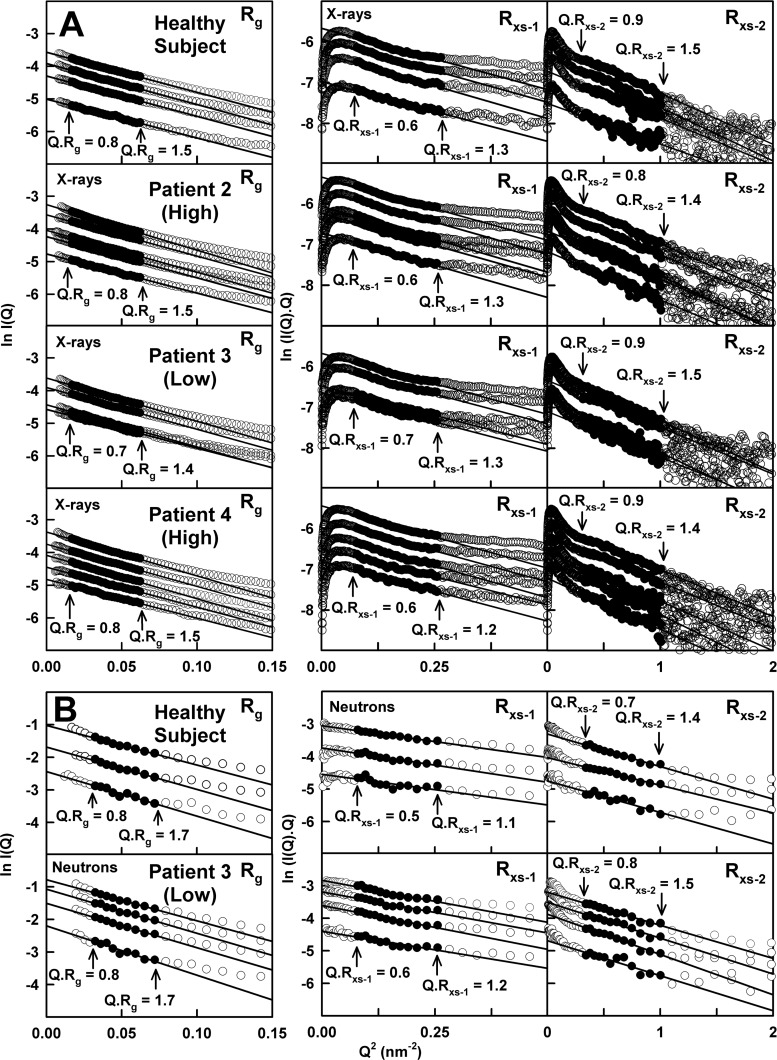
X-ray and neutron Guinier *R*_g_ and *R*_xs_ analyses for IgA1 (**A**) The X-ray scattering curves from bottom to top for 0.25, 0.51, 0.76 and 1.02 mg/ml of IgA1 from the healthy subject; 0.33, 0.49, 0.66, 0.99 and 1.33 mg/ml of IgA1 from patient 2; 0.36, 0.48, 0.72 and 0.97 mg/ml of IgA1 from patient 3; and 0.31, 0.47, 0.63, 0.94 and 1.26 mg/ml of IgA1 from patient 4, all measured at 20°C in PBS-137. The filled circles between the arrowed data points represent the *Q·R*_g_ and *Q·R*_xs_ ranges used to determine the *R*_g_ and *R*_xs_ values. The *Q*-ranges for the *R*_g_ values was 0.13–0.25 nm^−1^. The *Q*-ranges for the *R*_xs-1_ and *R*_xs-2_ values were 0.28–0.51 nm^−1^ and 0.56–1.01 nm^−1^ respectively. (**B**) The neutron scattering curves from bottom to top for 0.43, 0.85 and 1.7 mg/ml of IgA1 from the healthy subject and 0.5, 1.0, 1.6 and 2.2 mg/ml of IgA1 from patient 3 are shown for PBS-137 buffer in ^2^H_2_O at 20°C. The *Q* range used for the *R*_g_ values was 0.18–0.28 nm^−1^ and those for the *R*_xs-1_ and *R*_xs-2_ values were 0.28–0.51 nm^−1^ and 0.56–1.04 nm^−1^ respectively.

**Figure 5:**
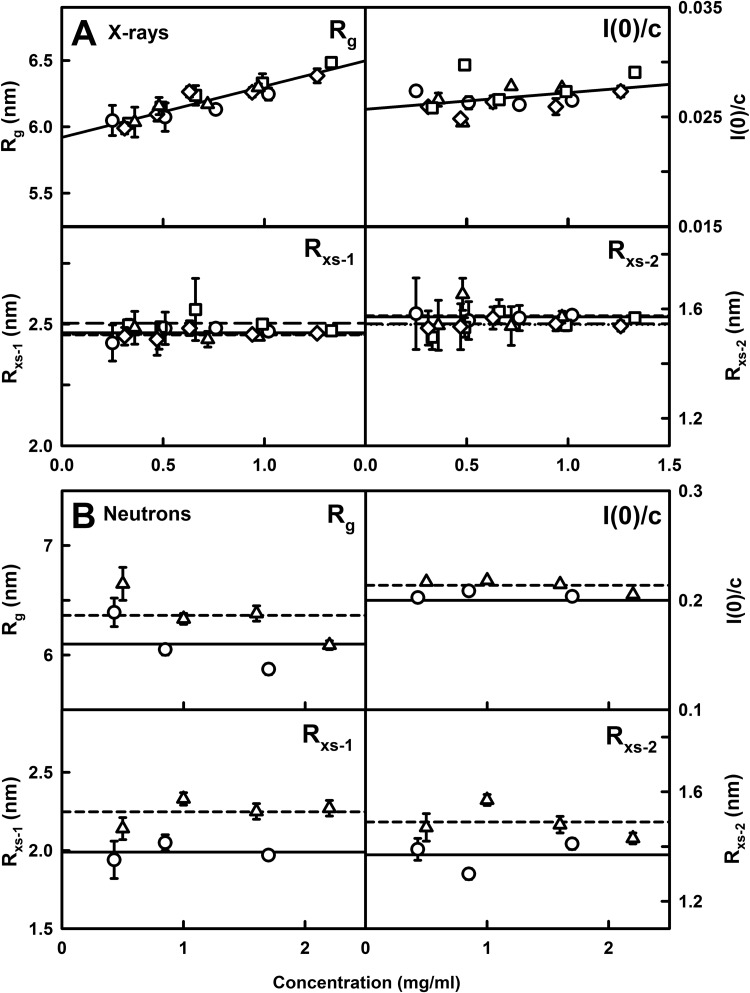
Concentration dependence of the Guinier values for IgA1 (**A**) The X-ray *R*_g_, *I(0)/c*, *R*_xs-1_ and *R*_xs-2_ values for the four IgA1 samples were each measured in quadruplicate and averaged to give the mean ± S.D. Error bars are shown only when visible. The values correspond to the healthy subject (○,—), patient 2 (□, − −), patient 3 (△, - - -) and patient 4 (⋄, ···). The *R*_g_ and *I(0)/c* data were fitted by linear regression, whereas the *R*_xs-1_ and *R*_xs-2_ data were fitted using their mean value. (**B**) The neutron *R*_g_, *I(0)/c*, *R*_xs-1_ and *R*_xs-2_ values for IgA1 each correspond to a single measurement in PBS-137 (^2^H_2_O). The fitted lines for IgA1 from healthy subject (○,—) and patient 3 (△, - - -) correspond to the mean values.

A total of 88 separate runs were measured by X-ray scattering for the four IgA1 samples at 20°C in concentration ranges between 0.12 and 1.33 mg/ml. Guinier analyses resulted in high-quality linear plots in three distinct regions of the *I(Q)* curves, as expected for antibodies, from which the *R*_g_, *R*_xs-1_ and *R*_xs-2_ values were obtained within satisfactory *Q·R*_g_ and *Q·R*_xs_ limits (Figure 4A; Table 1A). The lack of upward curvature in the Guinier plots at low *Q* values indicates the absence of non-specific aggregates. When plotted together, this extensive set of X-ray *R*_g_ values for the four IgA1 samples was sensitive to the concentration, unlike the sedimentation data. The X-ray *R*_g_ values of IgA1 increased with increasing concentration from 6.05 to 6.25 nm for healthy subject, 6.03 to 6.49 nm for patient 2, 6.04 to 6.30 nm for patient 3 and 5.99 to 6.39 nm for patient 4 (Figure 5A). Extrapolation to zero concentration resulted in very similar *R*_g_ values of 5.96 nm (healthy subject), 5.92 nm (patient 2), 5.93 nm (patient 3) and 5.92 nm (patient 4), once the noisier data from samples below 0.25 mg/ml were excluded (Table 1A). The corresponding *I(0)/c* values for IgA1 also increased with increase in concentration (Figure 5A). When combined with the *c(s)* analyses (Figure 3F), the increases in the *R*_g_ and *I(0)/c* values indicated weak non-covalent dimer formation that showed small increases with increased concentration. The *R*_xs-1_ and *R*_xs-2_ values of the four IgA1 samples were unchanged. The mean *R*_xs-1_ values were 2.46±0.06 nm, 2.50±0.06 nm, 2.46±0.05 nm and 2.46±0.04 nm and the mean *R*_xs-2_ values were 1.57±0.07 nm, 1.55±0.04 nm, 1.58±0.07 nm and 1.54±0.05 nm, for the healthy subject, patient 2, patient 3 and patient 4 respectively (Table 1A). These *R*_g_, *R*_xs-1_ and *R*_xs-2_ values were similar to the *R*_g_, *R*_xs-1_ and *R*_xs-2_ values of 6.20±0.13 nm, 2.20±0.26 nm and 1.56±0.16 nm respectively from limited data from seven runs for pooled human IgA1 [24]. However the concentration dependence seen in the much larger data set of 88 values of *R*_g_ and *I(0)/c* by the present study is novel. The X-ray data showed that, despite the wide variation in O-galactosylation, all four IgA1 samples exhibited closely similar extended overall structures. This outcome agreed with the sedimentation data (Figure 3F) and implied that the overall hinge conformation in the four IgA1 samples were similar.

For neutron scattering, the full-length IgA1 samples in PBS buffer in 100% ^2^H_2_O were analysed between 0.43 and 2.4 mg/ml. Only the neutron Guinier analyses for IgA1 from the healthy subject and patient 3 with high O-galactosylation revealed satisfactory linear *R*_g_, *R*_xs-1_ and *R*_xs-2_ fits as described above for X-rays (Figure 4B). The two IgA1 samples with low O-galactosylation showed non-specific aggregation and no Guinier analyses were possible. Aggregation of plasma glycoproteins in heavy water correlates with weaker solute–solvent hydrogen bonding interactions. The seven neutron *R*_g_ values for IgA1 from the healthy subject and patient 3 were unchanged with concentration at 20°C with average values of 6.10±0.26 nm and 6.36±0.23 nm (Table 1B). Because these *R*_g_ values were slightly larger than the X-ray values, this suggested that the two samples were not completely aggregate-free. The *R*_g_ and *I(0)/c* values did not reveal a concentration dependence, attributed to the availability of fewer data points (Figure 5B). The mean neutron *R*_xs-1_ values of 1.99±0.06 nm and 2.25±0.08 nm were similar and the mean *R*_xs-2_ values of 1.68±0.07 nm and 1.83±0.08 nm were similar also (Figure 5B). The neutron *R*_xs-1_ values were lower than the X-ray *R*_xs-1_ values and attributed to the near-invisible hydration shell by neutrons, whereas the neutron and X-ray *R*_xs-2_ values were more similar to each other (Table 1). The present Guinier data were similar to the previously reported neutron *R*_g_, *R*_xs-1_ and *R*_xs-2_ values for pooled IgA1 of 6.11±0.18 nm, 2.17±0.23 nm and 1.18±0.12 nm respectively [24].

**Figure 6:**
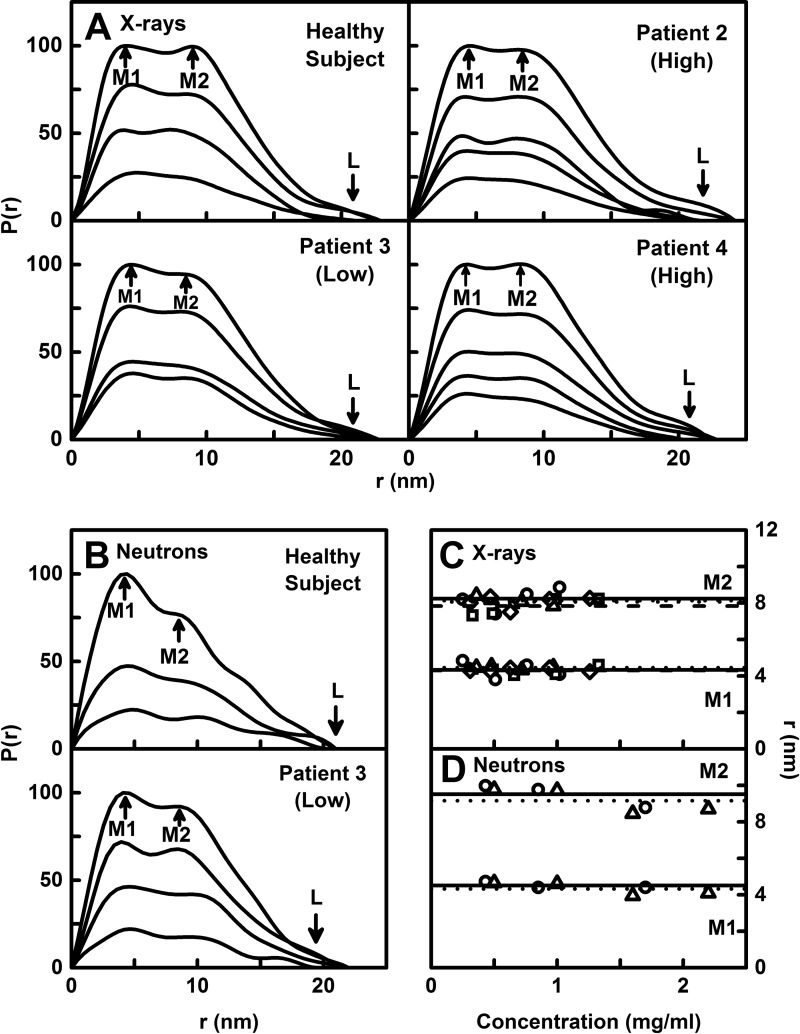
X-ray and neutron distance distribution analyses *P(r)* for IgA1 In the *P(r)* analyses, the peak maxima at *M1* and *M2* and the averaged maximum length of IgA1 at *L* are arrowed. (**A**) The X-ray *P(r)* curves for the four IgA1 samples are shown for concentrations between 0.25–1.33 mg/ml, based on the same curves shown in Figure 4(A). (**B**) The neutron *P(r)* curves for two IgA1 samples in PBS-137 (^2^H_2_O) buffer are shown for concentrations between 0.43–2.2 mg/ml based on the same curves shown in Figure 4(B). (**C**) The concentration dependences of the maxima *M1* and *M2* in the X-ray *P(r)* curves of (**A**) are shown. The symbols and lines for the healthy subject and three patient samples follow those in Figure 5(A). In all cases, the lines represent the mean values. (**D**) The corresponding concentration dependences of the maxima *M1* and *M2* in the neutron *P(r)* curves of (**B**) are shown, following the same symbols and lines as in Figure 5(B).

The distance distribution function *P(r)* provides structural information on full-length IgA1 in real space, this being equivalent to a histogram of all the distances between the atoms in IgA1. The *R*_g_ values from the X-ray *P(r)* analyses (result not shown) were similar to those from the X-ray Guinier analyses, showing that the two analyses were self-consistent. The maximum length *L* of IgA1 was determined from the value of *r* when the *P(r)* curve intersects zero at large *r*. The *L* values were 21.2±1.3 nm for the healthy subject, 22.1±1.6 nm for patient 2, 21.3±1.5 nm for patient 3 and 21.0±1.4 nm for patient 4 (arrowed; Figure 6A). The *L* values increased to 23 nm with increasing concentration for IgA1 from the healthy subject and the three patients, this being consistent with weak dimer formation. The two maxima *M1* and *M2* in the *P(r)* curves correspond to the most frequently occurring interatomic distances within the structure. The *M1* peak corresponded mostly to distances that arose within each of the three Fab and Fc regions (Figure 1A), whereas the *M2* peak corresponded mostly to distances between the Fab–Fab and Fab–Fc pairs. These two peaks were identified at approximately 4.5 nm and 8.0 nm respectively (Figure 6C). No concentration dependence in the positions of peaks *M1* and *M2* was observed and the four IgA1 samples showed similar *M1* and *M2* values, indicating that the IgA1 structure was unchanged with concentration or O-galactosylation. The current X-ray data resembled those for pooled human IgA1 but with a lower *M1* of 3.7 nm and a higher *M2* of 8.9 nm [24].

The neutron *P(r)* analyses of the two IgA1 samples in heavy water gave *R*_g_ values similar to their observed Guinier *R*_g_ values and did not change with increasing concentration. The mean neutron *L* value was 19.6±3.3 nm, being 21 nm for IgA1 from the healthy subject and 22 nm for IgA1 from patient 3 (Figure 6B) and were similar to the X-ray *L* values above. The two peaks *M1* and *M2* were identified at approximately 4.5 nm and 9 nm respectively in the neutron *P(r)* curves (Figures 6B and 6D). The positions of *M1* and *M2* did not change with concentration. Although the X-ray and neutron *M1* values were the same, the neutron *M2* values were higher than the X-ray *M2* values above. In summary, our extensive data sets for four IgA1 samples provided a sound basis for new molecular modelling of the IgA1 solution structure and revealed no conformational differences between IgA1 with different O-galactosylations.

### Neutron scattering modelling of PTerm455

Atomistic solution structures for IgA1 were determined by creating a large conformational library of structurally-accurate models, from which theoretical scattering curves were generated for comparison with the experimental scattering curve [28,29]. This atomistic modelling was initiated from the IgA Fab and Fc crystal structures (Experimental). The four principal unknowns in the IgA1 modelling were the conformation of (i) the two N-glycans at Asn^263^, (ii) the two hinges between the Fab and Fc regions, together with the two C-terminal tailpieces and their Asn^459^ N-glycans and the six O-glycans in the two hinges.

IgA1 was first modelled in terms of its structure without its tailpieces in order to focus on its Asn^263^ glycans and hinges. This modelling was made possible because good quality neutron scattering data were previously available for PTerm455, which is a tailpiece-deleted recombinant IgA1 that terminated at Pro^455^ (Figure 1C) [24].

(i) Firstly, two ‘initial’ T- and Y-shaped PTerm455 structures were generated and optimized using energy minimization (Experimental). These initial models contained the crystallographically-observed compact N-glycan conformations at Asn^263^ (PDB code 1OW0) in hydrogen-bond contact with the Fc surface. This conformation was denoted as NG0 (Supplementary Figure S1A). Two more alternate extended conformations were identified from MD simulations and denoted as NG1 and NG2 (Supplementary Figures S1C and S1D).

(ii) Secondly, randomization of the 20 backbone ϕ and ψ angles in the 21-residue hinge of PTerm455 generated trial PTerm455 structures, to which each of the NG0, NG1 and NG2 glycan conformers were added. Analysis of the resulting 172833 models showed that the lowest *R* factors corresponded to modelled *R*_g_ values that were close to the experimental neutron *R*_g_ value of 5.84±0.18 nm as desired [24]. These arose from the PTerm455 structures with the NG0 glycan (blue in Supplementary Figure S2). The curve fits thus did not favour the extended NG1 and NG2 N-glycan conformers.

### X-ray scattering modelling of full-length human IgA1

Trial full-length IgA1 structures were constructed from the best 36621 PTerm455 models (inset, Supplementary Figure S2). To these, four distinct glycosylated tailpiece structures TP1–4 were added to give 146484 full-length candidate IgA1 models (Supplementary Figure S3). Because the two tailpieces contribute less than 4% of the IgA1 structure, taking the best 21% of the PTerm455 models for this step is expected to provide an abundance of models that will include the best *R*-factors for intact IgA1. Many of the PTerm455 conformations excluded by the initial filter had very different *R*_g_ values from those selected, making it unlikely that these models would provide good fits to the X-ray curves.

**Figure 7:**
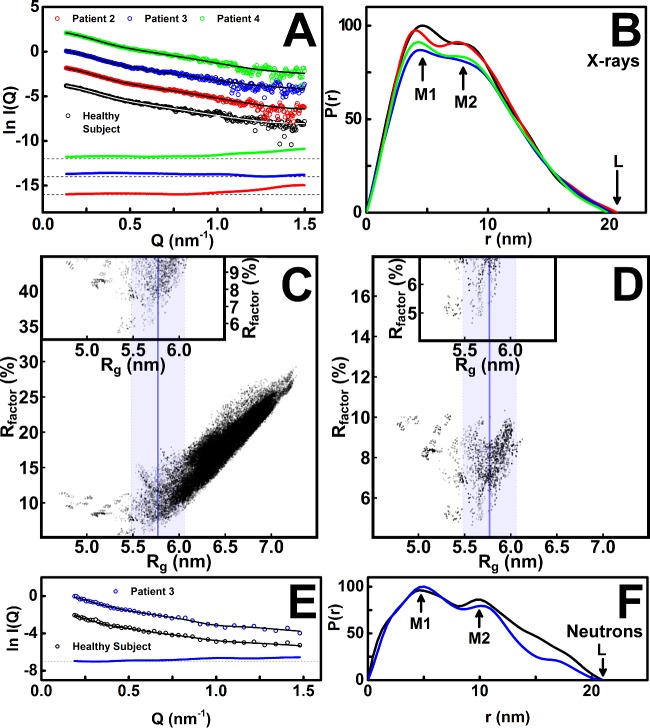
Outcome of the constrained modelling analyses of the full-length IgA1 solution structures including the tailpiece (**A**) The four experimental X-ray *I(Q)* curves for the healthy subject and patients 2, 3 and 4 extrapolated to zero concentration are shown (circles), together with the fitted *I(Q)* curves used for the calculation of the *P(r)* curves (white or black lines). Beneath these, the three difference curves obtained by subtracting the *I(Q)* curve for the healthy subject from that for each of the *I(Q)* curves for patients 2, 3 and 4 are shown in the same colour scheme and compared with a black dashed line as reference. (**B**) The four X-ray *P(r)* curves calculated from the four extrapolated *I(Q)* curves in (**A**) for the healthy subject and patients 2, 3 and 4 are shown, with the peak positions of *M1* and *M2* and the maximum dimension *L* labelled as arrowed. (**C**) The goodness-of-fit *R* factors for the calculated *I(Q)* curves for 146484 hydrated IgA1 structures compared with the extrapolated *I(Q)* curve for the healthy subject is plotted against the calculated *R*_g_ value for each hydrated model. These IgA1 structures do not contain the six O-glycans. The experimental *R*_g_ value of 5.77±0.04 nm (Table 2) is denoted by a vertical blue line, together with a coloured band that indicates the ±10% range of X-ray *R*_g_ values used for filtering of the best-fit models. In the inset, an expanded view is shown for the *R* factors below 10%. (**D**) The 3520 IgA1 structures showing *R* factors below 10% from (**C**) were modified by the addition of the six O-glycans to their two hinges. The *R* factors were recomputed using the extrapolated experimental *I(Q)* curve for the healthy subject. Other details follow (**C**). The inset expands the data set below 7%. (**E**) The two experimental neutron *I(Q)* curves for the healthy subject and patient 3 extrapolated to zero concentration are shown (circles), together with the fitted *I(Q)* curves used for the calculation of the *P(r)* curves (black lines). Beneath these, the difference curve obtained by subtracting the *I(Q)* curve for the healthy subject from that for patient 3 is shown. (**F**) The two neutron *P(r)* curves calculated from the extrapolated *I(Q)* curves for the healthy subject and patient 3 are shown.

Similar *s_20,w_* and X-ray and neutron *R*_g_ values were seen for all four IgA1 samples (Figures 3E, 5A and 5B). Because concentration dependences were visible in Figure 5(A) and trace amounts of dimers were detected in the *s_20,w_* analyses (Figure 3F), the full X-ray scattering curves were extrapolated to zero concentration to reduce any perturbations caused by these concentration effects. To follow the linear extrapolation of the *R*_g_ and *I(0)* values (Figures 5A and 5B), curve extrapolations were based on the best-fit regression lines plotted through the observed *I(Q)* values at each *Q* value for each curve in the concentration series. The subtraction of the extrapolated healthy subject X-ray and neutron curves from the extrapolated patient curves showed no significant features that would have indicated conformational differences between the four IgA1 samples (Figures 7A and 7E). The four X-ray and two neutron *P(r)* curves also showed close agreements (Figures 7B and 7F). When all four extrapolated X-ray scattering curves were modelled with the 172833 IgA1 models, all four *R-*factors compared with *R_g_* graphs were similar (Figure 7C). Interestingly, even though IgA1 has two long 23-residue hinges of maximum length 8.44 nm between the flanking α-carbon atoms of Pro^221^ and His^243^, the best fit IgA1 structures showed that these hinges were not maximally extended and had hinge lengths of 3.68 nm to 6.13 nm. These similarities between the four IgA1 samples showed that the variation in O-glycan contents had no effect on the length of the IgA1 hinge.

**Figure 8:**
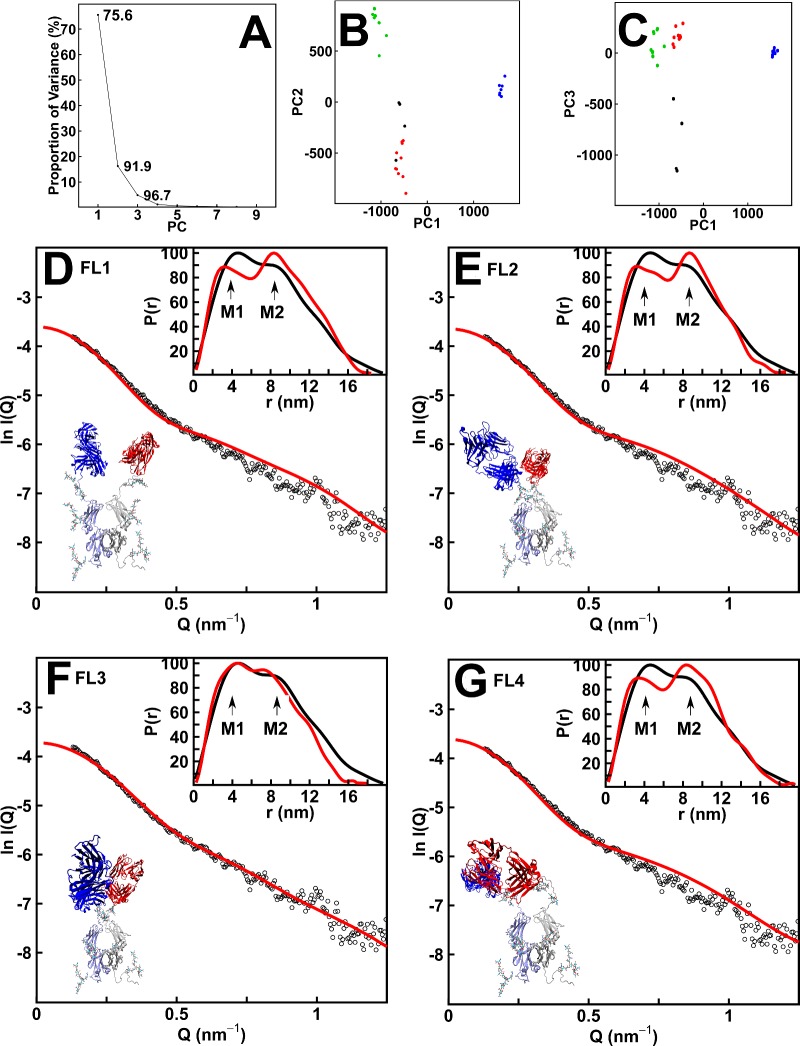
Principal component analysis of the best-fit full-length IgA1 models Models included in this analysis have *R* factors of less than 7% when compared with the healthy subject and three patient samples and their *R*_xs-1_ values were within 10% of the experimental values. (**A**) Conformational variability of the best-fit models from PC analysis. (**B** and **C**) Projections for each snapshot of the simulation trajectory shown as a function of PC1, PC2 and PC3. The resulting best-fit structures for full-length IgA1 are shown as FL1–4, in which black denotes FL1, red denotes FL2, green denotes FL3 and blue denotes FL4. (**D–G**) The curve fits for four representative structures for full-length IgA1 are shown for each of the four conformational clusters FL1–4 in that sequence. In each panel, the IgA1 structure is shown (Fab in red and blue; Fc in grey and light blue), together with the calculated *I(Q)* and *P(r)* curves in red, which are compared with the experimental data in black. The *R-*factor for each of the curves is given in Table 2.

Principal component analysis was used to determine the major conformations for IgA1. A total of 471 best-fit structures showed *R-*factors below 7% and *R*_g_ and *R*_xs-1_ values within 10% of the experimental values. The first three principal components (PCs; PC1, PC2 and PC3) accounted for 96.7% of the variation between these structures (Figure 8A). Hierarchical clustering of PC1, PC2 and PC3 identified four groups of best-fit structures FL1–4 (Figures 8B and 8C). Their X-ray *R*_g_ values agreed well with the experimental *R*_g_ value of 5.80 nm from the extrapolated scattering curve (Table 2). The *R-*factors for the 112 FL3 structures were the lowest at 5.4%–5.9% (Table 2). The visual agreement between the experimental and modelled *I(Q)* curves was the best for FL3, notably at large *Q* values between 0.6–1.2 nm^−1^ (Figure 8F). The modelled *P(r)* curve for FL3 showed the double peaks *M1* and *M2*, whereas those for FL1, FL2 and FL4 showed too pronounced a double peak (Figures 7B, 8D–8G). Thus the FL3 best-fit structures described best the average IgA1 solution structure as a compact Y-shape (Figure 8F).

**Table 2:** Summary of the X-ray, neutron and sedimentation modelling fits for full-length IgA1 including the O-glycans The mean *R*_g_ and *R* factor values are reported for each of the four clusters (FL1, FL2, FL3 and FL4) of candidate full-length IgA1 structures with three *O*-linked glycans added to Thr^225^, Thr^228^ and Ser^232^ in the hinge region (Figure 1C). Standard deviations are indicated as shown. The calculated sedimentation coefficients are shown also.

Cluster number	Number of best-fit models	Hydrated volume (nm^3^)	X-ray *R*_g_ (nm)	X-ray *R*_xs-1_ (nm)	X-ray *R*_xs-2_ (nm)	X-ray *R* factor (Healthy subject) (%)	X-ray *R* factor (Patient 2) (%)	X-ray *R* factor (Patient 3) (%)	X-ray *R* factor (Patient 4) (%)	Neutron *R*_g_ (nm)	Neutron *R* factor (Healthy subject) (%)	Neutron *R* factor (Patient 3) (%)	*s^0^_20,w_*(S)
FL1	54	246±2	5.84±0.06	2.49±0.09	1.25±0.04	6.8±0.5	6.9±0.5	6.4±0.4	6.4±0.4	5.48±0.05	8.1±0.2	10.3±0.3	6.41±0.11
FL2	152	246±3	5.73±0.04	2.54±0.07	1.31±0.04	6.8±0.5	6.8±0.5	6.4±0.3	6.4±0.3	5.40±0.04	8.2±0.2	10.9±0.3	6.56±0.71
FL3	112	248±3	5.41±0.09	2.51±0.10	1.38±0.13	5.4±0.4	5.5±0.4	5.9±0.3	5.9±0.3	5.14±0.07	10.1±0.6	12.8±0.5	6.84±0.48
FL4	153	244±3	5.77±0.09	2.46±0.05	1.33±0.05	6.3±0.8	6.3±0.8	6.0±0.5	6.0±0.5	5.41±0.07	8.4±0.5	10.8±0.6	6.69±0.55
Overall total	471	246±3	5.68±0.17	2.50±0.08	1.33±0.08	6.3±0.8	6.4±0.8	6.1±0.5	6.1±0.5	5.35±0.13	8.7±0.9	11.3±1.0	6.65±0.58
Experimental values	n.a.	273	5.80±0.04*	2.48±0.08	1.58±0.13	n.a.	n.a.	n.a.	n.a.	6.25±0.26	n.a.	n.a.	6.29±0.11

*In distinction to Table 1, the experimental *R*_g_ value of 5.80 nm was determined from Guinier analyses that were averaged from the four scattering curves extrapolated to zero concentration and used for calculation of the *R-*factors. This experimental *R*_g_ value is arrowed in Figure 7. The four individual experimental *R_g_* values were 5.77 nm, 5.86 nm, 5.78 nm and 5.78 nm for the healthy subject, patient 2, patient 3 and patient 4 respectively.

n.a., not applicable.

Comparison of the most representative FL1–4 structures closest to the centroid of each cluster showed that the Fab regions were conformationally similar in the FL2 and FL3 structures, differing only by a displacement of the two Fab regions relative to the Fc region. In the most representative FL1 and FL4 structures, the Fab arrangements were very similar to each other (Figure 8B). The median FL1–4 structures showed different Fc orientations (Figures 8D–8G). Thus the modelling was unable to define a single Fc orientation relative to the Fab regions, although all four of FL1–4 indicated an overall Y-shaped structure for the two Fab regions relative to Fc.

To evaluate the effect of O-galactosylation levels in IgA1, six O-glycans at Thr^225^, Thr^228^ and Ser^232^ were added to the two hinges of the 471 FL1–4 best-fit structures (Experimental). These contribute ∼1% of the residues in IgA1. For FL3, the 112 *R-*factors were 4.8%–6.2% for the healthy control, which were improved compared with that of 6.5% for models without O-glycans (Figure 7D). Similar improvements were seen for FL1, FL2 and FL4 and the other three samples. Although the addition of additional scattering density at the IgA1 hinge improved the fits, the similarity of the four IgA1 scattering curves showed that these were unaffected by altered *O*-galactosylation levels.

As an independent control of the X-ray fits, the 471 FL1–4 best-fit models were used to compute the *s^0^_20,w_* values for comparison with the experimental value of 6.29±0.11 S. The mean *s^0^_20,w_* values were 6.41 S, 6.56 S, 6.84 S and 6.69 S for FL1, FL2, FL3 and FL4 respectively (Table 2), with a mean value of 6.56±0.58 S. Given that the experimental and calculated values generally agree within ± 0.21 S [28], it was concluded that all four FL1–4 best-fit structures were consistent with the experimental *s^0^_20,w_* values.

**Figure 9:**
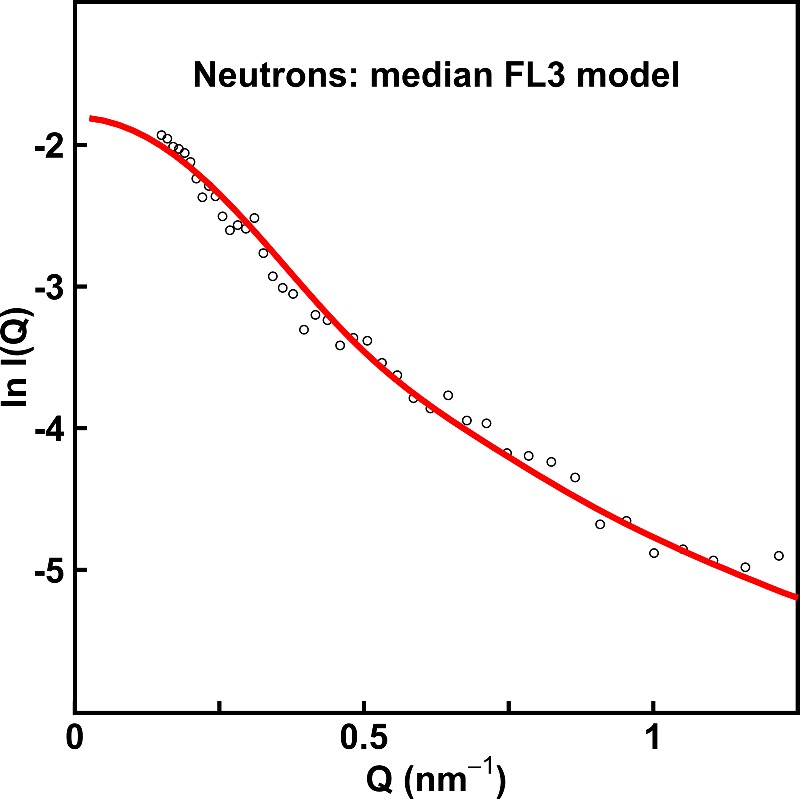
Neutron curve fit using the best-fit FL3 IgA1 structure The neutron fit corresponding to the X-ray fit of Figure 8(F) is shown. The calculated *I(Q)* curve is shown as a red line, which is compared with the experimental data points in black. A flat incoherent scattering correction of 1.5% of *I(0)* was applied to the calculated curve to allow for the proton content of the IgA1 neutron sample.

As a further independent control of the X-ray fits, the neutron data for IgA1 were also fitted to the unhydrated 146484 full-length IgA1 models. The 100 best fit models with the lowest *R*-factors of 8% displayed *R*_g_ values close to 5.5 nm, indicating that the mean experimental *R*_g_ value of 6.25±0.26 nm corresponded to slightly aggregated IgA1 in heavy water. The best neutron curve fit showed poorer signal-noise ratios than the X-ray fits of Figure 8 (Figure 9). Nonetheless the fair agreement with the best-fit X-ray models and the satisfactory neutron *R* factors (Table 2) showed that the neutron fits were compatible with the X-ray fits.

## DISCUSSION

**Figure 10:**
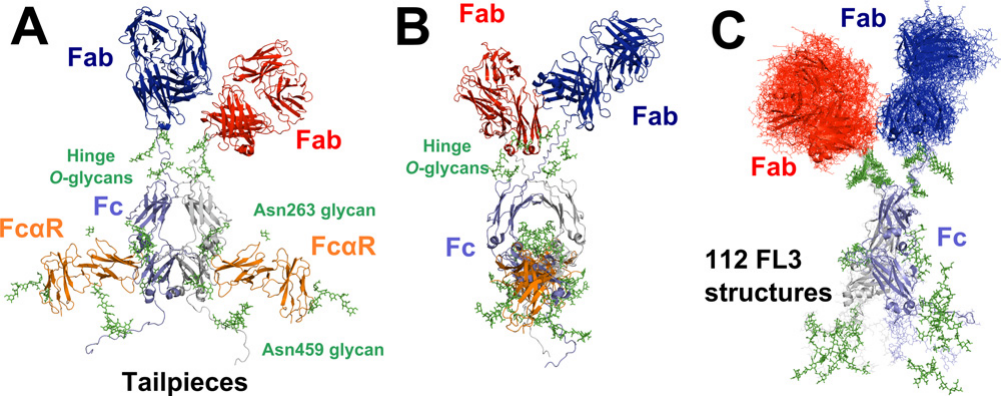
Binding of the FcαR receptor to a best-fit full-length IgA1 model (**A** and **B**) Face-on and side-on views of the Fc region in a best-fit Y-shaped IgA1 structure are shown, taken from the median of the FL3 cluster of Figure 8. The two views are rotated by 90° about a vertical axis, with the two FcαR sites on the Fc region bound to two FcαR receptors (orange: PDB code 1OW0). The two Fab regions are shown in red and blue and the Fc region is shown in grey and light blue. The N-glycans at Asn^263^ and Asn^459^ in the Fc region and tailpiece respectively and the O-glycans at Thr^225^, Thr^228^ and Ser^232^ in the hinge are shown as green sticks. This structure is also shown in the Supplemental Movie S1. (**C**) View of the 112 IgA1 models in the FL3 cluster, all of which were superimposed on the Fc region. The view was orientated to show the spread of the tailpiece and 2×112 best-fit Fab conformations. The median structure of the FL3 cluster (frame 62) is shown as a cartoon ribbon; the other 111 models are shown as lines.

Our abundant X-ray scattering data from improved instrumentation for four human IgA1 samples with a range of hinge *O*-galactosylations, together with a new modelling procedure, resulted in atomistic Y-shaped solution structures for IgA1 (Figure 10C). Our asymmetric extended IgA1 solution structures in combination with the crystal structure of the Fc–FcαR receptor complex [26] show that the Fab regions in IgA1 were positioned well away from its Fc region (Figures 10A and 10B). The exposed Fc region is thus able to interact readily with its FcαR receptor (Supplementary Movie S1). During this investigation, IgA1 was found to be prone to non-specific aggregation or self-association and its relationship with the level of hinge O-galactosylation may be relevant to the onset of IgAN.

### Solution structure of monomeric human IgA1

The three new analytical ultracentrifugation and X-ray and neutron scattering analyses for four IgA1 samples, including three from IgAN patients, have notably extended our understanding of the IgA1 solution structure.

(i) In the present study, 17 ultracentrifugation runs were performed on IgA1 from the four donors. Here, our use of *c(s)* distribution plots in SEDFIT now revealed small amounts of non-covalent IgA1 dimer, in distinction to our previous distribution analyses in DCDT+ sedimentation software were unable to reveal dimers [62]. The mean IgA1 *s^0^_20,w_* values of 6.29 S (Table 1) agreed well with previous values of 6.15–6.20 S from DCDT+ [62].

(ii) Recent instrumental advances enabled the abundant measurement of 88 X-ray scattering curves in a single beam session on the synchrotron (or 880 curves if time frames are included). This wealth of data permitted the detailed study of four different human IgA1 samples with varied O-glycan content in physiological buffer. A newly-observed concentration-dependence of the *R*_g_ values was seen and this was explained by the minor IgA1 dimer formation seen by ultracentrifugation. Accordingly, to eliminate dimer contributions to the scattering curves, it was important to extrapolate these to zero concentration prior to the structural modelling. In distinction, our previous X-ray scattering study reported too few scattering runs to identify concentration effects [24]. Also previously, pooled IgA1 samples were used which were likely to be more polydisperse than our present samples. Despite these differences, similar experimental X-ray *R*_g_, *R*_xs-1_ and *R*_xs-2_ values of 5.93, 2.48 and 1.58 nm were measured for all four IgA1 samples (Table 1), compared with previous values of 6.20, 2.20 and 1.56 nm respectively [24]. The higher previous X-ray *R*_g_ value is attributable to these concentration effects (Figure 5A).

(iii) Our 13 neutron curves gave *R*_g_, *R*_xs-1_ and *R*_xs-2_ values of 6.25, 2.13 and 1.44 nm (Table 1), compared with previous values of 6.11, 2.17 and 1.18 nm respectively [24]. Because the neutron *R*_g_ values were slightly higher than their corresponding X-ray values, which were unexpected given the solute–solvent contrast in use, the new data clarified that IgA1 in heavy water buffer showed minor aggregation and this may be relevant to IgAN (see below).

Our more detailed scattering modelling resulted in improved Y-shaped IgA1 structures that replaced our previous T-shaped structures [24]. The current modelling benefitted from extensive experimental data, compared with the limited X-ray and neutron data sets from before (Figures 8C and 8F of [24]). The FL3 cluster of best-fit models gave *R-*factors of 5.4%–5.9% (Table 2), compared with a high *R-*factor of 10.9% for our previous model in 1999 [24]. The discrepancy in shape between 1999 and the current modelling was attributed to both the concentration dependence in the X-ray *R*_g_ values and the slight aggregation present in the neutron curves, neither of which was known in 1999. This outcome illustrates the importance of acquiring high-quality data sets prior to performing modelling fits. When compared with the original IgA EM studies [63–66], although some earlier studies did not differentiate between the IgA1 and the IgA2 isotypes, Y-shaped IgA structures were in fact observed that were flexible at the hinge between the Fab and Fc regions. The IgA2 isotype contains a shorter hinge region and is presumed to be less flexible [62]. The scattering modelling of dimeric IgA1 and secretory IgA1 also indicated Y-shaped structures and not T-shaped ones [36,67]. Overall, Y-shaped IgA1 structures appear as its most widely accepted consensus structure.

New insight into the variable O-galactosylated IgA1 hinges of IgA1 resulted from the overall solution structures for the four IgA1 samples. The similarity of the hinges was shown by the similar concentration dependences of the *R*_g_ and *I(0)* values (Figures 5A and 5B), the similar *s^0^_20,w_* values and the similar *M1* and *M2* values (Figures 3E, 6C and 6D). The difference curves between the three patient and healthy subject IgA1 samples also showed no discernible differences (Figures 7A and 7E). Atomistic modelling for each of the four samples revealed that IgA1 has mostly (but not fully) extended hinge structures. If the three NeuNAc.Gal.GalNAc O-glycans at Thr^225^, Thr^228^ and Ser^232^ were added to the hinges (Figure 10), the three O-glycans occupy significant surface areas of the two hinges. The O-glycans may act as potential spacers. If so, reductions in O-glycan contents may alter the flexibility of the IgA1 hinges, leading to the entanglement of the hinges of different IgA1 molecules and may pre-dispose IgA1 to its aggregation.

### Interaction of human IgA1 with its FcαR receptor

Our new IgA1 solution structure clarifies its functional interaction with FcαR receptors (CD89) on monocyte, eosinophil, macrophage and neutrophil cell surfaces [68]. When the IgA1 models from the FL3 cluster were superimposed on to the crystal structure of the Fc–FcαR complex, the full-sized IgA1 structure readily accommodated two FcαR molecules (Figures 10A and 10B) [26]. This observation agrees with the 1:2 stoichiometry for IgA1 binding to FcαR observed by surface plasmon resonance [69]. The 1:2 IgA1–receptor complex will form at a cell surface if there is a sufficient surface density of FcαR molecules. IgA1 binding to FcαR receptors will trigger phagocytosis, antibody-dependent cell-mediated cytotoxicity and a respiratory burst in neutrophils [70]. Given that the serum concentration of IgA1 is 3 mg/ml (19 μM) with 90% in the monomeric form [70] and the dissociation constants *K*_D_ for IgA1 binding to FcαR are low between 0.18 and 0.43 μM [69], it is deduced that plasma IgA1 will bind to surface-bound FcαR molecules. Due to the moderately fast on- and off-rates of the FcαR–IgA1 binding reaction (2×10^5^ M·s^−1^ and 0.04 s^−1^ respectively) [69], rapid sampling of free IgA1 would occur. In summary, it is expected that a Y-shaped arrangement of Fab regions in IgA1 that coats a pathogenic surface will result in a surface of IgA1 Fc regions that is in the right orientation to bind to many FcαR receptors to trigger appropriate immune responses.

Interestingly, IgA–receptor binding is very different from IgG–receptor and IgE–receptor binding [71]. First, FcαR binds between the Cα2 and the Cα3 domains of IgA1 away from the hinge (Figure 10). In contrast, FcγR binds to IgG at the top of the C_H_2 domain near the bottom of the hinge. Like IgG, FcεR binds at the Cε2/Cε3 domain interface of IgE where the Cε2 domain of IgE is equivalent to the IgG hinge. Second, the FcRs (Fc receptors) generally have two-domain receptor structures, with membrane-distal and membrane-proximal extracellular domains. The membrane-distal domain of FcαR binds to IgA1, but, in contrast, the membrane-proximal domains of FcγR and FcεR bind to IgG and IgE respectively. Third, IgA–FcαR complexes exhibit a 1:2 binding stoichiometry, whereas IgG–FcγR and IgE–FcεR complexes exhibit 1:1 binding stoichiometries. This is explained by the adoption of asymmetric conformations by IgG and IgE such that the binding of one FcγR and FcεR receptor molecule blocks the binding of a second receptor molecule. No such blocking occurs in IgA1. The three O-glycans in each of the two IgA1 hinges may be important for FcαR binding by hindering the movement of the Fab regions that might otherwise block the FcαR sites in the Fc region. The IgA1 tailpieces have no defined structures, but are likely to be extended into solution and will not block FcαR binding. Whereas IgG and IgE antibodies have to reposition their Fab regions away from the FcR-coated cell surface in order to permit receptor binding, there is no such requisite displacement of the Fab regions in IgA1 when FcαR binds. Our recent atomistic scattering modelling of the solution structures of the IgG1 and IgG4 sub-classes extends this understanding [72,73]. Their asymmetric structures enable FcγR to bind to the Fc region of IgG1 without obstructions with the Fab regions, whereas this was not possible for FcγR binding to IgG4, in which the hinge is shorter and the Fab regions will obstruct binding [72,73]. Thus the IgG1 solution structure is already preformed to permit FcγR binding.

### Role of IgA1 monomers in IgAN

IgAN is a significant cause of kidney disease across the world. Central to the pathogenesis of this common glomerulonephritis is the formation of large circulating IgA1-containing immune complexes that have a pre-disposition to deposit in the glomerulus and trigger inflammation and scarring with consequent loss of kidney function. One of the key observations in a population of IgAN patients is the presence of poorly galactosylated IgA1 in both the serum and the glomerular immune deposits. This finding is consistently reproduced in populations of different geographic origin, although there can be a considerable spread in IgA1 O-glycan content in individual patients with kidney biopsy-proven IgAN [9,18–20]. Changes in the hinge O-glycans have previously been hypothesized to alter the conformation of IgA1, thereby promoting IgA1 immune complex formation in IgAN through IgA1 aggregation and self-association [74] and the generation of neoepitopes, leading to the generation of IgA and IgG hinge-region specific auto-antibodies. Even though these immune complexes predominantly consist of polymeric IgA1, an understanding of the structural impact of the hinge O-glycans on IgAN firstly requires an evaluation of their impact on monomeric IgA1.

Our experience of four IgA1 samples with variable O-glycan contents suggests that the instability of IgA1 monomers contributes to the development of IgAN, rather than conformational changes in IgA1. The symmetric profiles in size-exclusion chromatography indicated that most IgA1 eluted as stable monomers in physiological buffer (Figure 2). Nonetheless, freezing causes IgA1 to aggregate. The low amounts (below 5%) of non-covalent IgA1 dimers seen by ultracentrifugation (Figure 3) indicated a minor tendency for the monomer to self-associate. Because heavy water is a known promoter of protein self-association (because solute–solvent hydrogen bonds are weaker in heavy water), it was of interest that IgA1 in heavy water buffer showed slight aggregation for the two samples with higher O-glycan contents and stronger aggregation for the two samples with low O-glycan contents. Because these self-association events do not appear for human IgG1 and IgG4 [72,73], the IgA1 solution structure shows a pre-disposition to self-associate. If this is confirmed in further biophysical investigations, this will clarify the molecular basis of IgAN.

### Postscript: atomistic modelling of antibodies

Atomistic scattering modelling offers unique views of antibody solution structure. Only three crystal structures are available for one human and two murine intact IgG antibodies [75,76]. Even with these, it is not assured that a single view of an antibody immobilized in a crystal lattice will represent its solution structure; there is a common expectation that antibodies display many conformations in solution. The previous automated atomistic modelling of IgA1 in 1999 was the first analysis of its type for X-ray and neutron scattering and resulted in the first deposition of a scattering-determined molecular structure in the Protein Data Bank (PDB code 1IGA) [24]. In that analysis, 12000 trial randomized IgA1 structures were generated from a MD procedure applied to the hinge peptide. The Fab and Fc regions in these first IgA1 structures were constructed using homology (comparative) modelling based on crystal structures for a murine IgA Fab region and a human IgG1 Fc region. The previous modelling assumed that the IgA1 structure would be 2-fold symmetric about the Fc region; however, this assumption is now no longer justified.

In the present study, the atomistic modelling of IgA1 benefitted from both the IgA1 Fab and the Fc crystal structures [26,34] and the development of a new methodology SASSIE to generate randomized IgA1 structures [30,31,58]. Because earlier crystallography and scattering fits revealed that human and mouse IgG antibodies were not necessarily 2-fold symmetric [72,73,75,76], the assumption of symmetry was discarded for IgA1. During model generation, a 14-fold increase from 12000 [24] to 172833 randomized hinge conformations in IgA1 was generated by Monte Carlo variations of 20 θ and ψ angles in the IgA1 hinge. This dihedral Monte Carlo approach is rapid and sampled 50000 structures in less than an hour on a single CPU. Because the hinge and Fab/Fc regions were considered together when creating each trial IgA1 structure, steric overlaps were handled better; poor models were now discarded at their creation, rather than later. In addition, the combination of rapid global structure sampling simulations and force field representations of trial structures allowed more detailed computational studies of IgA1 (Supplementary Figures S1–S3). The quality of the curve fits in a *Q* range out to 1.25 nm^−1^ (Figure 8F) with a *R-*factor as low as 4.8% is improved compared with that from 1999 in a *Q* range out to 2.0 nm^−1^ with an *R*-factor of 5.4% (Figure 9B in [24]). Both the 1999 and the current analyses resulted in similarly extended IgA1 structures, although the detail in the current study makes the conclusion in the present study of Y-shaped structures more reliable. The success of the present IgA1 modelling indicates that atomistic modelling for antibody structures has high potential for structure–function studies of antibody–ligand complexes, as well as for other systems.

## Abbreviations

Fab: fragment antigen-binding
Fc: fragment crystallisable
FcR: Fc receptor
FL: full-length
Gal: galactose
GalNAc: *N*-acetylgalactosamine
HAA: *Helix aspersa*
IgAN: IgA nephropathy
*I*(*Q*): scattering curve
MD: molecular dynamics
NeuNAc: sialic acid
NG: N-glycan
PC: principal component
*Q*: scattering vector
*R*_g_: radius of gyration
TP: tail-piece

## References

[1] Woof J.M., Mestecky J. (2005). Mucosal immunoglobulins. Immunol. Rev..

[2] van Egmond M., van Garderen E., van Spriel A.B., Damen C.A., van Amersfoort E.S., van Zandbergen G., van Hattum J., Kuiper J., van de Winkel J.G. (2000). FcαRI-positive liver Kupffer cells: reappraisal of the function of immunoglobulin A in immunity. Nat. Med..

[3] Woof J.M., Russell M.W. (2011). Structure and function relationships in IgA. Mucosal Immunol..

[4] Hiemstra P.S., Gorter A., Stuurman M.E., van Es L.A., Daha M.R. (1987). Activation of the alternative pathway of complement by human serum IgA. Eur. J. Immunol..

[5] Hiemstra P.S., Biewenga J., Gorter A., Stuurman M.E., Faber A., van Es L.A., Daha M.R. (1988). Activation of complement by human serum IgA, secretory IgA and IgA1 fragments. Mol. Immunol..

[6] Roos A., Bouwman L.H., van Gijlswijk-Janssen D.J., Faber-Krol M.C., Stahl G.L., Daha M.R. (2001). Human IgA activates the complement system via the mannan-binding lectin pathway. J. Immunol..

[7] Roos A., Rastaldi M.P., Calvaresi N., Oortwijn B.D., Schlagwein N., van Gijlswijk-Janssen D.J., Stahl G.L., Matsushita M., Fujita T., van Kooten C., Daha M.R. (2006). Glomerular activation of the lectin pathway of complement in IgA nephropathy is associated with more severe renal disease. J. Am. Soc. Nephrol..

[8] Barratt J., Feehally J. (2005). IgA nephropathy. J. Am. Soc. Nephrol..

[9] Suzuki H., Kiryluk K., Novak J., Moldoveanu Z., Herr A.B., Renfrow M.B., Wyatt R.J., Scolari F., Mestecky J., Gharavi A.G., Julian B.A. (2011). The pathophysiology of IgA nephropathy. J. Am. Soc. Nephrol..

[10] Boyd J.K., Cheung C.K., Molyneux K., Feehally J., Barratt J. (2012). An update on the pathogenesis and treatment of IgA nephropathy. Kidney Int..

[11] Baenziger J., Kornfeld S. (1974). Structure of the carbohydrate units of IgA1 immunoglobulin. II. Structure of the O-glycosidically linked oligosaccharide units. J. Biol. Chem..

[12] Mattu T.S. (1998). The glycosylation and structure of human serum IgA1, Fab and Fc regions and the role of N-glycosylation on Fcα receptor interactions. J. Biol. Chem..

[13] Royle L., Roos A., Harvey D.J., Wormald M.R., van Gijlswijk-Janssen D., Redwan el-R.M., Wilson I.A., Daha M.R., Dwek R.A., Rudd P.M. (2003). Secretory IgA N- and O-glycans provide a link between the innate and adaptive immune systems. J. Biol. Chem..

[14] Yoo E.M., Morrison S.L. (2005). IgA: an immune glycoprotein. Clin. Immunol..

[15] Hiki Y., Tanaka A., Kokubo T., Iwase H., Nishikido J., Hotta K., Kobayashi Y. (1998). Analyses of IgA1 hinge glycopeptides in IgA nephropathy by matrix-assisted laser desorption/ionization time-of-flight mass spectrometry. J. Am. Soc. Nephrol..

[16] Hiki Y., Odani H., Takahashi M., Yasuda Y., Nishimoto A., Iwase H., Shinzato T., Kobayashi Y., Maeda K. (2001). Mass spectrometry proves under-O-glycosylation of glomerular IgA1 in IgA nephropathy. Kidney Int.

[17] Tarelli E., Smith A.C., Hendry B.M., Challacombe S.J., Pouria S. (2004). Human serum IgA1 is substituted with up to six O-glycans as shown by matrix assisted laser desorption ionisation time-of-flight mass spectrometry. Carbohydr. Res..

[18] Lai K.N. (2012). Pathogenesis of IgA nephropathy. Nat. Rev. Nephrol..

[19] Barratt J., Smith A.C., Molyneux K., Feehally J. (2007). Immunopathogenesis of IgAN. Semin. Immunopathol..

[20] Mestecky J., Raska M., Julian  B.A., Gharavi A.G., Renfrow M.B., Moldoveanu Z., Novak L., Matousovic K., Novak J. (2013). IgA nephropathy: molecular mechanisms of the disease. Annu. Rev. Pathol..

[21] Dourmashkin R.R., Virella G., Parkhouse R.M. (1971). Electron microscopy of human and mouse myeloma serum IgA. J. Mol. Biol..

[22] Feinstein A., Munn E.A., Richardson N.E. (1971). The three-dimensional conformation of γM and γA globulin molecules. Ann. N.Y. Acad. Sci..

[23] Munn E.A., Feinstein A., Munro A.J. (1971). Electron microscope examination of free IgA molecules and of their complexes with antigen. Nature.

[24] Boehm M.K., Woof J.M., Kerr M.A., Perkins S.J. (1999). The Fab and Fc fragments of IgA1 exhibit a different arrangement from that in IgG: a study by X-ray and neutron solution scattering and homology modelling. J. Mol. Biol..

[25] Correa A., Trajtenberg F., Obal G., Pritsch O., Dighiero G., Oppezzo P., Buschiazzo A. (2013). Structure of a human IgA1 Fab fragment at 1.55 Å resolution: potential effect of the constant domains on antigen-affinity modulation. Acta Crystallogr. D Biol. Crystallogr..

[26] Herr A.B., Ballister E.R., Bjorkman P.J. (2003). Insights into IgA-mediated immune responses from the crystal structures of human FcαRI and its complex with IgA1-Fc. Nature.

[27] Ramsland P.A., Willoughby N., Trist H.M., Farrugia W., Hogarth P.M., Fraser J.D., Wines B.D. (2007). Structural basis for evasion of IgA immunity by Staphylococcus aureus revealed in the complex of SSL7 with Fc of human IgA1. Proc. Natl. Acad. Sci. U.S.A..

[28] Perkins S.J., Okemefuna A.I., Nan R., Li K., Bonner A. (2009). Constrained solution scattering modelling of human antibodies and complement proteins reveals novel biological insights. J.R. Soc. Interface.

[29] Perkins S.J., Nan R., Li K., Khan S., Abe Y. (2011). Analytical ultracentrifugation combined with X-ray and neutron scattering: experiment and modelling. Methods.

[30] Curtis J.E., Raghunandan S., Nanda H., Krueger S. (2012). SASSIE: A program to study intrinsically disordered biological molecules and macromolecular ensembles using experimental scattering restraints. Comput. Phys. Commun..

[31] Clark N.J., Zhang H., Krueger S., Lee H.J., Ketchem R.R., Kerwin B., Kanapuram S.R., Treuheit M.J., McAuley A., Curtis J.E. (2013). Small-angle neutron scattering study of a monoclonal antibody using free-energy constraints. J. Phys. Chem. B.

[32] Allen A.C., Bailey E.M., Barratt J., Buck K.S., Feehally J. (1999). Analysis of IgA1 O-glycans in IgA nephropathy by fluorophore-assisted carbohydrate electrophoresis. J. Am. Soc. Nephrol..

[33] Gomes M.M., Suzuki H., Brooks M.T., Tomana M., Moldoveanu Z., Mestecky J., Julian B.A., Novak J., Herr A.B. (2010). Recognition of galactose-deficient O-glycans in the hinge region of IgA1 by N-acetylgalactosamine-specific snail lectins: a comparative binding study. Biochemistry.

[34] Correa A., Trajtenberg F., Obal G., Pritsch O., Dighiero G., Oppezzo P., Buschiazzo A. (2013). Structure of a human IgA1 Fab fragment at 1.55 Å resolution: potential effect of the constant domains on antigen-affinity modulation. Acta Crystallogr..

[35] Perkins S.J. (1986). Protein volumes and hydration effects: the calculation of partial specific volumes, neutron scattering matchpoints and 280-nm absorption coefficients for proteins and glycoproteins from amino acid sequences. Eur. J. Biochem..

[36] Bonner A., Furtado P.B., Almogren A., Kerr M.A., Perkins S.J. (2008). Implications of the near-planar solution structure of human myeloma dimeric IgA1 for mucosal immunity and IgA nephropathy. J. Immunol..

[37] Laue T.M., Shah B.D., Ridgeway T.M., Pelletier S.L., Harding S.E., Rowe A.J., Horton J.C. (1992). Computer-aided interpretation of analytical sedimentation data for proteins. In Analytical Ultracentrifugation in Biochemistry and Polymer Science.

[38] Schuck P. (1998). Sedimentation analysis of non-interacting and self-associating solutes using numerical solutions to the Lamm equation. Biophys. J..

[39] Schuck P. (2000). Size-distribution analysis of macromolecules by sedimentation velocity ultracentrifugation and Lamm equation modelling. Biophys. J..

[40] Narayanan T., Diat O., Bösecke P. (2001). SAXS and USAXS on the high brilliance beamline at the ESRF. Nucl. Instrum. Methods Phys. Res. Section A.

[41] Heenan R.K., Rogers S.E., Turner D., Terry A.E., Treadgold J., King S.M. (2011). Small angle neutron scattering using Sans2d. Neutron News.

[42] Glatter O., Kratky O. (1982). Small Angle X-ray Scattering.

[43] Pilz I., Kratky O., Licht A., Sela M. (1973). Shape and volume of anti-poly(D-alanyl) antibodies in the presence and absence of tetra-D-alanine as followed by small-angle X-ray scattering. Biochemistry.

[44] Semenyuk A.V., Svergun D.I. (1991). GNOM–a program package for small-angle scattering data-processing. J. Appl. Crystallogr..

[45] Gomes M.M., Wall S.B., Takahashi K., Novak J., Renfrow M.B., Herr A.B. (2008). Analysis of IgA1 N-glycosylation and its contribution to FcαRI binding. Biochemistry.

[46] Jo S., Kim T., Iyer V.G., Im W. (2008). CHARMM-GUI: a web-based graphical user interface for CHARMM. J. Comput. Chem..

[47] Jo S., Song K.C., Desaire H., MacKerell A.D., Im W. (2011). Glycan reader: automated sugar identification and simulation preparation for carbohydrates and glycoproteins. J. Comput. Chem..

[48] MacKerell A.D., Bashford D., Bellott M., Dunbrack R.L., Evanseck J.D., Field M.J., Fischer S., Gao J., Guo H., Ha S. (1998). All-atom empirical potential for molecular modeling and dynamics studies of proteins. J. Phys. Chem. B.

[49] Mackerell A.D., Feig M., Brooks C.L. (2004). Extending the treatment of backbone energetics in protein force fields: limitations of gas-phase quantum mechanics in reproducing protein conformational distributions in molecular dynamics simulations. J. Comput. Chem..

[50] Guvench O., Hatcher E.R., Venable R.M., Pastor R.W., Mackerell A.D. (2009). CHARMM Additive all-atom force field for glycosidic linkages between hexopyranoses. J. Chem. Theory Comput..

[51] Hatcher E., Guvench O., Mackerell A.D. (2009). CHARMM additive all-atom force field for acyclic polyalcohols, acyclic carbohydrates and inositol. J. Chem. Theory Comput..

[52] Raman E.P., Guvench O., Mackerell A.D. (2010). CHARMM additive all-atom force field for glycosidic linkages in carbohydrates involving furanoses. J. Phys. Chem. B.

[53] Tanner D.E., Chan K.Y., Phillips J.C., Schulten K. (2011). Parallel generalized born implicit solvent calculations with NAMD. J. Chem. Theory. Comput..

[54] Phillips J.C., Braun R., Wang W., Gumbart J., Tajkhorshid E., Villa E., Chipot C., Skeel R.D., Kalé L., Schulten K. (2005). Scalable molecular dynamics with NAMD. J. Comput. Chem..

[55] Grant B.J., Rodrigues A.P.C., ElSawy K.M., McCammon J.A., Caves L.S.D. (2006). Bio3D: an R package for the comparative analysis of protein structures. Bioinformatics.

[56] Novak J., Julian B.A., Tomana M., Mestecky J. (2008). IgA glycosylation and IgA immune complexes in the pathogenesis of IgA nephropathy. Semin. Nephrol..

[57] Takahashi K., Wall S.B., Suzuki H., Smith, IV A.D., Hall S., Poulsen K., Kilian M., Mobley J.A., Julian B.A., Mestecky J., Novak J., Renfrow M.B. (2010). Clustered O-glycans of IgA1: defining macro- and microheterogeneity by use of electron capture/transfer dissociation. Mol. Cell. Proteomics.

[58] Wright D.W., Perkins S.J. (2015). SCT: a suite of programs for comparing atomistic models to small angle scattering data. J. Appl. Crystallogr.

[59] Perkins S.J. (2001). X-ray and neutron scattering analyses of hydration shells: a molecular interpretation based on sequence predictions and modelling fits. Biophys. Chem..

[60] Perkins S.J., Weiss H. (1983). Low-resolution structural studies of mitochondrial ubiquinol:cytochrome c reductase in detergent solutions by neutron scattering. J. Mol. Biol..

[61] Kerr M.A. (1990). The structure and function of human IgA. Biochem. J..

[62] Furtado P.B., Whitty P.W., Robertson A., Eaton J.T., Almogren A., Kerr M. A, Woof J.M., Perkins S.J. (2004). Solution structure determination of monomeric human IgA2 by X-ray and neutron scattering, analytical ultracentrifugation and constrained modelling: a comparison with monomeric human IgA1. J. Mol. Biol..

[63] Svehag S.E., Bloth B. (1970). Ultrastructure of secretory and high-polymer immunoglobulin A of human and rabbit origin. Science.

[64] Munn E.A., Feinstein A., Munro A.J. (1971). Electron microscope examination of free IgA molecules and of their complexes with antigen. Nature.

[65] Feinstein A., Munn E.A., Richardson N.E. (1971). The three-dimensional conformation of γM and γA globulin molecules. Annu. Rev. Acad. Sci..

[66] Dourmashkin R.R., Virella G., Parkhouse R.M. (1971). Electron microscopy of human and mouse myeloma serum IgA. J. Mol. Biol..

[67] Bonner A., Almogren A., Furtado P.B., Kerr M.A., Perkins S.J. (2009). Location of secretory component on the Fc edge of dimeric IgA1 reveals insight into the role of secretory IgA1 in mucosal immunity. Mucosal Immunol.

[68] Monteiro R.C., Kubagawa H., Cooper M.D. (1990). Cellular distribution, regulation and biochemical nature of an Fc alpha receptor in humans. J. Exp. Med..

[69] Herr A.B., White C.L., Milburn C., Wu C., Bjorkman P.J. (2003). Bivalent binding of IgA1 to FcαRI suggests a mechanism for cytokine activation of IgA phagocytosis. J. Mol. Biol..

[70] Delves P.J., Martin S.J., Burton D.R., Roitt I.M. (2011). Roitt's Essential Immunology.

[71] Woof J.M., Burton D.R. (2004). Human antibody–Fc receptor interactions illuminated by crystal structures. Nat. Rev. Immunol..

[72] Rayner L.E., Hui G.K., Gor J., Heenan R.K., Dalby P.A., Perkins S.J. (2014). The Fab conformations in the solution structure of human IgG4 restricts access to its Fc region: implications for functional activity. J. Biol. Chem..

[73] Rayner L.E., Hui G.K., Gor J., Heenan R.K., Dalby P.A., Perkins S.J. (2015). The solution structures of two human IgG1 antibodies show conformational stability and accommodate their C1q and FcγR ligands. J. Biol. Chem..

[74] Kokubo T., Hiki Y., Iwase H., Tanaka A., Toma K., Hotta K., Kobayashi Y. (1998). Protective role of IgA1 glycans against IgA1 self-aggregation and adhesion to extracellular matrix proteins. J. Am. Soc. Nephrol..

[75] Saphire E.O., Parren P.W., Pantophlet R., Zwick M.B., Morris G.M., Rudd P.M., Dwek R.A., Stanfield R.L., Burton D.R., Wilson I.A. (2001). Crystal structure of a neutralizing human IgG against HIV-1: a template for vaccine design. Science.

[76] Harris L.J., Larson S.B., Skaletsky E., McPherson A. (1998). Comparison of the conformations of two intact monoclonal antibodies with hinges. Immunol. Rev..

